# Cannabinoid receptor 2 plays a pro-tumorigenic role in non-small cell lung cancer by limiting anti-tumor activity of CD8^+^ T and NK cells

**DOI:** 10.3389/fimmu.2022.997115

**Published:** 2023-01-09

**Authors:** Arailym Sarsembayeva, Melanie Kienzl, Eva Gruden, Dusica Ristic, Kathrin Maitz, Paulina Valadez-Cosmes, Ana Santiso, Carina Hasenoehrl, Luka Brcic, Jörg Lindenmann, Julia Kargl, Rudolf Schicho

**Affiliations:** ^1^ Division of Pharmacology, Otto Loewi Research Center, Medical University of Graz, Graz, Austria; ^2^ Diagnostic and Research Institute of Pathology, Medical University of Graz, Graz, Austria; ^3^ Division of Thoracic and Hyperbaric Surgery, Department of Surgery, Medical University of Graz, Graz, Austria; ^4^ BioTechMed, Graz, Austria

**Keywords:** CB_1_, CB_2_, cannabinoid receptors, non-small cell lung cancer, tumor microenvironment, CD8^+^ T cells, NK cells, immunotherapy

## Abstract

Cannabinoid (CB) receptors (CB_1_ and CB_2_) are expressed on cancer cells and their expression influences carcinogenesis in various tumor entities. Cells of the tumor microenvironment (TME) also express CB receptors, however, their role in tumor development is still unclear. We, therefore, investigated the role of TME-derived CB_1_ and CB_2_ receptors in a model of non-small cell lung cancer (NSCLC). Leukocytes in the TME of mouse and human NSCLC express CB receptors, with CB_2_ showing higher expression than CB_1_. In the tumor model, using CB_1_- (CB_1_^-/-^) and CB_2_-knockout (CB_2_^-/-^) mice, only deficiency of CB_2_, but not of CB_1_, resulted in reduction of tumor burden vs. wild type (WT) littermates. This was accompanied by increased accumulation and tumoricidal activity of CD8^+^ T and natural killer cells, as well as increased expression of programmed death-1 (PD-1) and its ligand on lymphoid and myeloid cells, respectively. CB_2_^-/-^ mice responded significantly better to anti-PD-1 therapy than WT mice. The treatment further increased infiltration of cytotoxic lymphocytes into the TME of CB_2_^-/-^ mice. Our findings demonstrate that TME-derived CB_2_ dictates the immune cell recruitment into tumors and the responsiveness to anti-PD-1 therapy in a model of NSCLC. CB_2_ could serve as an adjuvant target for immunotherapy.

## Introduction

Cannabinoid (CB) receptors CB_1_ and CB_2_ are widely found in human tumor tissue and are well-known to influence the growth of tumor cells (1). However, whether they act as tumor promotors or suppressors, and whether CB receptors located in cancer cells or/and in immune cells of the tumor microenvironment (TME) are involved in tumor progression, is less clear. In particular, CB receptors could significantly influence the development of lung cancer, as suggested by previous studies of non-small cell lung cancer (NSCLC) (2, 3). Some studies show that agonists of CB_1_ and/or CB_2_ attenuate the carcinogenic potential in lung cancer cells (2, 4–6), and reduce tumor growth in immunodeficient (7) and FVB/N mice (8), however, other studies report the opposite. For instance, CB_1_/CB_2_ agonist tetrahydrocannabinol (THC) may promote proliferation of lung cancer cells (9) and the growth of breast cancer *in vivo* (10). In addition, silencing of CB_2_ in lung cancer cells reportedly decreases their proliferation, migration, and invasion (3). A number of studies on the prognostic value of CB expression revealed discrepant findings based on the cancer type (reviewed in (11)). While some articles described high expression of CB_1_/CB_2_ receptors in human samples of NSCLC correlating with prolonged survival (2), others described a positive correlation of CB_2_ expression with increased tumor size and pathological grading of NSCLC (3), indicating a complex and still unclear role of CB receptors in NSCLC.

CB_1_ and CB_2_ receptors are part of the endocannabinoid system (ECS), acting in concert with their endogenous ligands (endocannabinoids) and enzymes for synthesis and degradation of these ligands (12, 13). CB_1_ is abundantly expressed in the central nervous system (14), but is also detectable in peripheral tissues including the immune system (15, 16). The majority of immune cells express CB_1_ at low levels, and its expression is generally affected by the activation status and cell type, as well as the presence of immune stimuli and endocannabinoids (17). In contrast, CB_2_ is highly expressed in immune cells, and controls functions such as proliferation, migration, activity, cytokine release, antigen presentation, and antibody production (15, 18). The receptor has previously been described for its immunosuppressive behavior (15, 19). For instance, in plaque-forming cell assays in mouse splenocytes (which measure the capacity of the spleen cells to mount a primary antibody response to sheep red blood cells), THC could directly inhibit the cells *via* CB_2_ (20). In addition, the endocannabinoid anandamide suppresses release of pro-inflammatory cytokines like IL-2, TNF-α and IFN-γ from activated human peripheral T-lymphocytes, acting primarily through CB_2_ (21). These effects can be mimicked by the CB_2_ agonist JWH-015, and blocked by the CB_2_ antagonist SR144528 (22). Cannabinoids have been reported to reduce natural killer cell (NK) activity, thus, *in vivo* administration of THC in male Swiss mice results in inhibition of splenic NK cytolytic activity without altering proliferation of splenocytes (23). Also, in human NK cells, THC has been demonstrated to reduce cytolytic activity (reviewed in Braile et al. (24)). CB_2_ has previously been suggested to play a key role in suppressing immune activity in cancer, a concept supported by Zhu et al., who showed that CB_2_ controls tumor immunity of lung cancer by increasing the levels of Th_2_ cytokines like IL-10 and TGF, and by downregulating the Th_1_ cytokine IFN-γ (10).

Based on their well-described impact on immune cells, CB receptors could significantly influence immune cell behavior and regulatory components of immune activity, including inhibitory checkpoint proteins like programmed death-1 (PD-1) and its ligand PD-L1, within the TME. PD-1 is an inhibitory receptor expressed on T cells after antigen stimulation, while PD-L1 is found on tumor cells and antigen presenting cells (25). Particularly, in NSCLC, the PD-1/PD-L1 axis has emerged as a successful target for the use of immune checkpoint inhibitors (ICI). However, limited response rates and resistance have hampered their success (26), warranting the discovery of new targets to boost ICI therapy. In this regard, clinical trials using combination therapies of ICIs with anti-angiogenic agents, chemotherapy, ataxia telangiectasia and Rad3-related **(**ATR) kinase and mitogen-activated protein kinase kinase (MEK) inhibitors, have been conducted or are still ongoing (reviewed in Blach et al. (26)).

In the present study, we investigated whether CB receptors located in the TME control tumor growth and influence susceptibility to ICI treatment. To investigate our hypothesis, we used a mouse model of NSCLC, in which immunocompetent wild type (WT) and CB_1_-knockout (CB_1_^-/-^) or CB_2_-knockout (CB_2_^-/-^) mice received a subcutaneous (s.c.) injection of syngeneic lung adenocarcinoma cells (KP cells (27)), thus creating a tumor model with TME cells that either express or lack CB receptor. We report that tumors in CB_2_^-/-^ mice are smaller than in their WT littermates, and that CB_2_^-/-^ mice respond better to anti-PD-1 therapy, indicating that CB_2_ expression in the TME is a critical determinant of immune suppression in this NSCLC model.

## Results

### Tumor and TME cells express CB receptors *in situ*, and blockade of CB_2_, and not CB_1_, inhibits tumor growth in a murine NSCLC model

**Figure 1:**
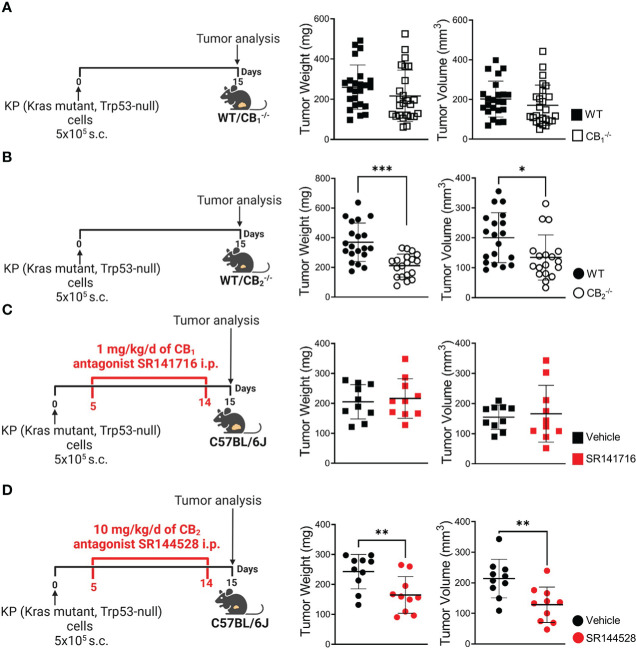
Blockade of CB_2_, but not CB_1_, inhibits tumor growth in a mouse model of NSCLC. **(A)** Experimental design: CB_1_^-/-^ mice and wild type (WT) littermates were subcutaneously (s.c.) injected with 5x10^5^ KP (Kras mutant, Trp53-null) lung adenocarcinoma cells on day 0. On day 15, tumors were measured *ex vivo* and harvested for analysis. Data indicate mean values ± SD from three pooled independent experiments. n= 23-25. **(B)** Experimental design: CB_2_^-/-^ mice and WT littermates were s.c. injected with 5x10^5^ KP lung adenocarcinoma cells on day 0. On day 15, tumors were measured *ex vivo* and collected for analysis. Data indicate mean values ± SD from two pooled independent experiments. n= 18-20. **(C, D)** Experimental design: C57BL/6J WT mice were s.c. injected with 5x10^5^ KP lung adenocarcinoma cells on day 0. Five-days post-inoculation, KP cell tumor-bearing mice started receiving intraperitoneal (i.p.) injections of either **(C)** 1 mg/kg/d of CB_1_ antagonist SR141716 or **(D)** 10 mg/kg/d of CB_2_ antagonist SR144528 (or vehicle). On day 15, tumor weight and volume were measured *ex vivo*. One representative experiment is shown. Data indicate mean values ± SD, n= 9-10. All statistical differences were evaluated by using unpaired student`s *t*-test **(A–D)**. *p < .05; **p < .01; ***p < .001. *NSCLC*, non-small cell lung cancer.

As the role of TME-derived CB receptors in lung cancer has not yet been investigated, we aimed to identify whether TME host cells lacking CB_1_ or CB_2_ would influence primary tumor growth. After injecting KP cells s.c. into the flanks of CB_1_^-/-^, CB_2_^-/-^, and WT mice, *ex vivo* measurement of tumor weight and volume demonstrated that tumor burden of CB_1_^-/-^ mice did not differ from WTs in our mouse model (**Figure 1A**). In contrast, mice devoid of CB_2_ showed more than 50% reduction in both tumor weight and volume, as compared to WT littermates (**Figure 1B**). We then investigated whether pharmacological blockade of CB receptors in tumor-bearing C57BL/6J mice could replicate findings obtained in knockout mice using previously tested doses of CB_1_ antagonist SR141716 (28, 29) and CB_2_ antagonist SR144528 (29, 30). As a result, treatment with CB_1_ antagonist SR141716 had no effect on both tumor weight and volume (**Figure 1C**), whereas tumor-bearing C57BL/6J mice treated with CB_2_ antagonist SR144528 showed a significant reduction in tumor weight and volume as compared to vehicle-treated animals (**Figure 1D**).

**Figure 2:**
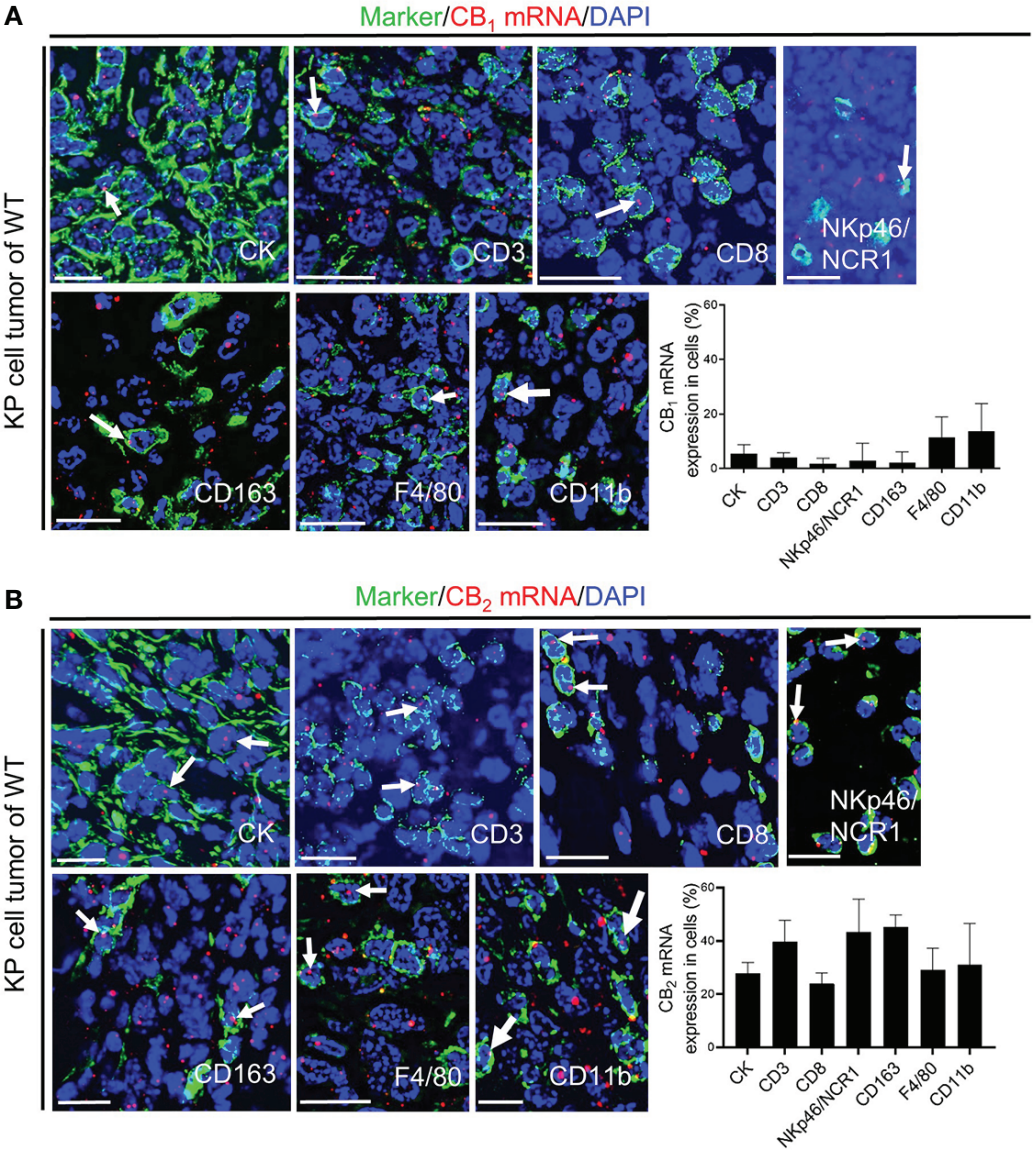
CB_1_ and CB_2_ mRNA in tumor cells and immune cells of the TME. **(A, B)** *In situ* hybridization (ISH)/immunofluorescence (IF) of tumor/immune cells in KP cell tumor sections from wild type mice. **(A)** The graph demonstrates the percentages of co-localization of CB_1_ mRNA positive signals with tumor cells (cytokeratin-stained, CK^+^ cells; ~ 5%) and leukocytes of the TME, such as CD3^+^ T cells (~ 4%), CD8^+^ T cells (~ 3%), NKp46/NCR1^+^ cells (natural killer, NK cells; ~ 14%), CD163^+^ M2 macrophages (~ 7%), F4/80^+^ M1 and M2 macrophages (~ 11%), and CD11b^+^ myeloid cells (~ 14%). **(B)** The graph shows the percentages of co-localization of CB_2_ mRNA signals with tumor cells (~ 25%) and tumor-infiltrating immune cells, including CD3^+^ T cells (~ 39%), CD8^+^ T cells (~ 24%), NKp46/NCR1^+^ NK cells (~ 43%), CD163^+^ M2 macrophages (~ 43%), F4/80^+^ M1 and M2 macrophages (~ 29%), and CD11b^+^ myeloid cells (~ 29%). Arrows denote CB_1_ or CB_2_ ISH mRNA signals within tumor and immune cells. Calibration bars=20 μm. Data indicate mean values +SD; n=3 (sections from three different tumors, 30-150 cells counted per section). *TME*, tumor microenvironment.

To further investigate the role of CB receptors in the TME, we identified mRNA expression of these receptors in tumor cells and infiltrating immune cells *in situ*. We used *in situ* hybridization (ISH) technique with specific probes against CB_1_ and CB_2_ mRNA in combination with immunofluorescence (IF). Dual ISH-IF analysis displayed CB_1_ expression in cancer cells as well as immune cells of the TME, but to a far lesser extent than expression of CB_2_ (**Figure 2A**). Around 25% of tumor cells (which positively stained for cytokeratin) co-localized with CB_2_ mRNA (**Figure 2B**). Within the TME, we detected CB_2_ mRNA expression in CD3^+^ T cells, CD8^+^ T cells, NKp46/NCR1^+^ cells, CD163^+^ or F4/80^+^ macrophages, and CD11b^+^ cells. Co-localizations ranged between ~20-40% (**Figure 2B**).

**Figure 3:**
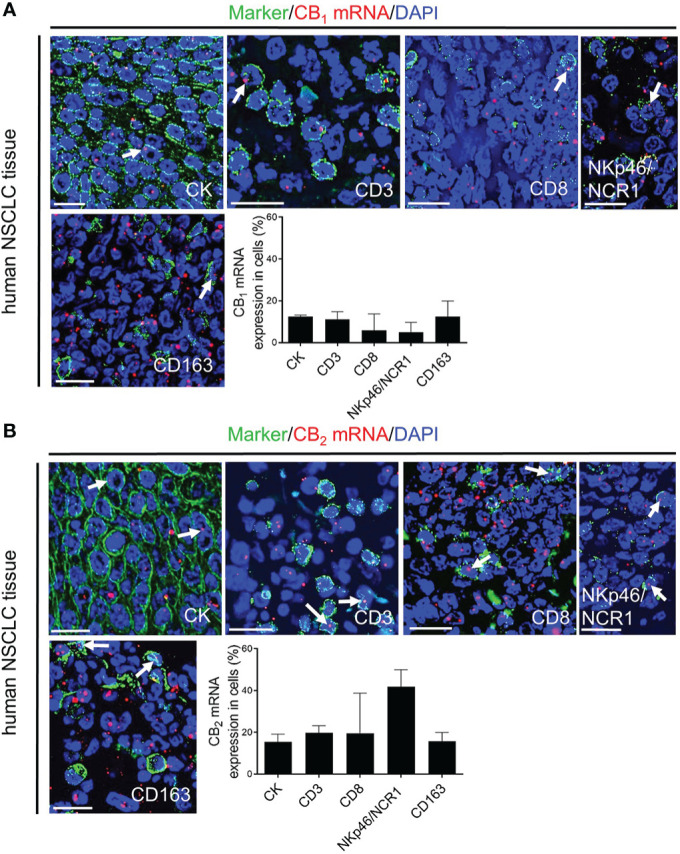
*In situ* hybridization (ISH)/immunofluorescence (IF) of human NSCLC tissue sections **(A, B)** Representative fluorescence microscopy images of human NSCLC tissue sections. The graphs show the percentages of co-localization of CB_1_ and CB_2_ mRNA signals with tumor cells (cytokeratin-stained, CK^+^ cells) as well as tumor-infiltrating immune cells (CD3^+^ T cells, CD8^+^ T cells, NKp46/NCR1^+^ NK cells, and CD163^+^ M2 macrophages). Arrows indicate CB_1_ and CB_2_ ISH signals within tumor and immune cells of the TME. Calibration bars = 20 μm. Data indicate mean values +SD. n=3 (tumor sections from three different patients with NSCLC were used for quantification, 30-150 cells counted per section). *NSCLC*, non-small cell lung cancer; *NK*, natural killer cells; *TME*, tumor microenvironment.

Since several studies described CB receptor expression in tumors of NSCLC patients (2, 3, 7), we stained sections of human lung cancer tissues to assess the distribution of CB_1_ and CB_2_ receptors in tumor cells and infiltrating immune cells, and also applied flow cytometry in freshly resected NSCLC tissues. In line with our mouse data, CB_1_ and CB_2_ expression were not only seen in lung cancer cells, but also in infiltrated immune cells, such as CD3^+^ T and CD8^+^ T cells, NKp46/NCR1^+^or CD56^+^ NK cells, and CD163^+^ macrophages. Expression of CB_2_ was generally higher than that of CB_1_ (**Figures 3A, B**, **S2A**).

These results indicate that CB_1_ and CB_2_ is expressed in both tumor and tumor-infiltrated immune cells, however, only deletion of CB_2_ on host cells or systemic blockade of CB_2_, but not of CB_1_, results in a reduction of tumor burden. To validate our results from the KP cell tumor model, we used Lewis lung carcinoma (LLC1) cells in CB_2_^-/-^ vs. WT mice and identified that tumor burden was significantly reduced in CB_2_^-/-^ mice when compared to WT mice (**Figure S2C**).

### Tumor reduction exclusively relies on deletion of CB_2_ in TME host cells

**Figure 4:**
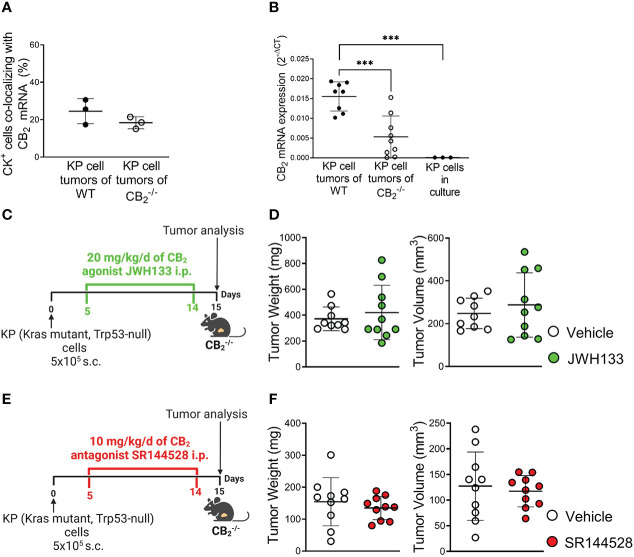
Tumor reduction exclusively relies on deletion of CB_2_ in TME host cells. **(A)** The graph depicts the percentage of CB_2_ mRNA positive cells co-localizing with cytokeratin-stained (CK^+^) tumor cells in mouse KP cell tumors, as evaluated by ISH-IF. Data indicate mean values ± SD. n=3/group (sections from three different tumors, 75-150 cells counted per section). **(B)** Relative CB_2_ mRNA expression as measured by qPCR in lysates from KP cell tumors from WT and CB_2_^-/-^ mice, as well as KP cells in culture. Data indicate mean values ± SD. n≥8/group; n=3 (consecutive passages of KP cells). **(C–F)** Experimental design: CB_2_^-/-^ mice were subcutaneously (s.c.) injected with 5x10^5^ KP (Kras mutant, Trp53-null) lung adenocarcinoma cells on day 0. For ten days, CB_2_^-/-^ mice were treated intraperitoneally (i.p.) with either **(C)** 20 mg/kg/d of CB_2_ agonist JWH133 or **(E)** 10mg/kg/d of CB_2_ antagonist SR144528 (or vehicle). Tumor weight and volume were measured at the end of the experiment *ex vivo* on day 15. One representative experiment is shown. Data indicate mean values ± SD. n≥9. Statistical differences were evaluated by using unpaired student`s *t*-test **(A, D, F)** or one-way ANOVA with Tukey’s multiple comparison test **(B)**. ***p<.001. *TME*, tumor microenvironment; *ISH/IF*, *in situ* hybridization and immunofluorescence; *WT*, wild type.

According to dual ISH-IF, we found that besides immune cells, around 20-25% of tumor cells in human NSCLC (**Figures 3B**, **S2A**) and mouse tumor (**Figures 4A**, **S2D**) tissue co-localized with CB_2_ mRNA. According to RT-qPCR, tumors of WT mice showed higher levels of CB_2_ mRNA than those from CB_2_^-/-^ mice, because host cells, such as immune cells infiltrating the TME in CB_2_^-/-^ mice, are devoid of CB_2_ expression (**Figure 4B**). KP cells in culture cells expressed minimal levels of CB_2_ (**Figures 4B**, **S2D**). We confirmed the specificity of our CB_2_ PCR primers by absence of CB_2_ mRNA expression in spleen tissue of CB_2_^-/-^ mice in comparison to WT mice (**Figure S2E**).

To address the role of CB_2_-expressing KP cells on tumor growth *in situ*, we pharmacologically activated or blocked CB_2_ in tumor-bearing CB_2_^-/-^ mice using a CB_2_ agonist (JWH133) (**Figure 4C**) or CB_2_ antagonist (SR144528) (**Figure 4E**) at previously published doses (29, 31). The results revealed that activation or inhibition of CB_2_ in tumor cells of tumor-bearing CB_2_^-/-^ mice had no effect on tumor weight and volume (**Figures 4D, F**), indicating that the tumor reduction we observed in the CB_2_^-/-^ mice solely depended on CB_2_, expressed in cells of the TME.

### Knockout of CB_2_ in cells of the TME favors an anti-carcinogenic immune cell profile and enhances CD8^+^ T and NK cell activity

**Figure 5:**
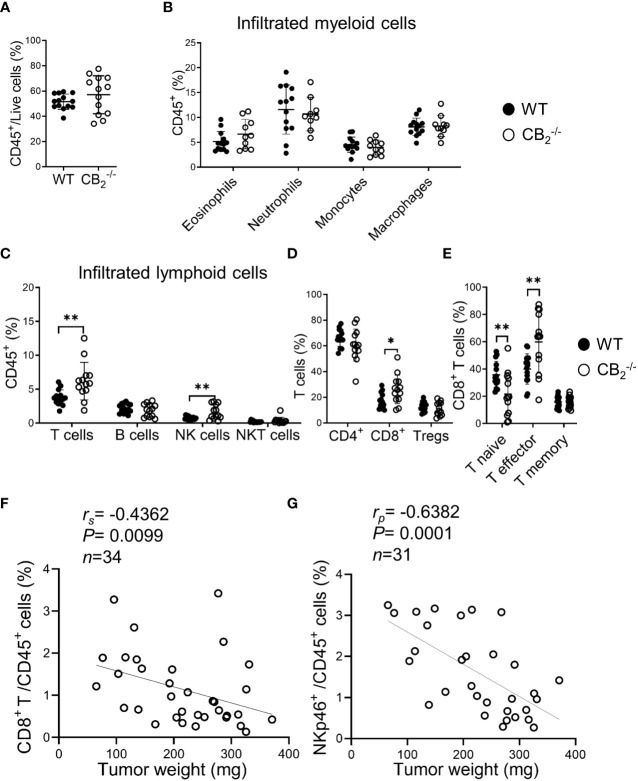
Knockout of CB_2_ in cells of the TME favors an anti-carcinogenic immune cell profile. **(A–E)** Flow cytometric analysis of single cell suspensions from KP cell tumors. Data indicate mean values ± SD from two pooled independent experiments. n≥10. Detailed information on immune cell markers is provided in **Figure S1**. Statistical differences were evaluated by using unpaired student`s *t*-test **(A)**, multiple *t*-tests **(B–E)**. **(F, G)** The percentages of tumor-infiltrating CD8^+^ T (CD45^+^/CD3^+^/CD8^+^) and NK (CD45^+^/CD3^-^/CD19^-^/NKp46^+^) cells (out of CD45^+^ cells) were plotted against tumor weights from CB_2_^-/-^ mice. Data were pooled from four independent experiments. n=31-34. Correlation of samples was assessed using Spearman (r_s_) and Pearson (r_p_) correlation coefficients after testing for normality. *p < .05; **p < .01. *NK*, natural killer cells, *NKT*, natural killer T cells; *TME*, tumor microenvironment; *Tregs*, regulatory T cells; *WT*, wild type.

To determine the immune cell profile in tumors of CB_2_^-/-^ and WT mice, we used flow cytometry and identified changes in infiltration of immune cells and their subtypes, observing a significant shift of lymphoid cell populations in CB_2_^-/-^ as compared to WT mice (gating strategies shown in **Figures S1A–C**). There were no significant differences in the infiltration of CD45^+^ leukocytes and myeloid cells between tumors of CB_2_^-/-^ and WT mice (**Figures 5A, B**, **S3A**). We, however, observed an increased infiltration of T cells (CD3^+^), NK cells (NKp46^+^), and CD8^+^ T cells (**Figures 5C, D**, **S3B–D**), but no differences in infiltration of CD4^+^ T and regulatory T cells (Tregs) into tumors of CB_2_^-/-^ mice vs. WTs (**Figure 5D**). Within the CD8^+^ T cell population, the number of effector CD8^+^ T cells increased while naïve CD8^+^ T cells decreased (**Figures 5E**, **S3E**), indicating that CD8^+^ T cells from CB_2_^-/-^, but not from WT mice, were primed to become effector cells. Percentages of infiltrating CD8^+^ T (**Figure 5F**) as well as NK cells (**Figure 5G**) negatively correlated with tumor weight in CB_2_^-/-^ mice. Furthermore, no significant changes in lymphoid immune cell composition, including T, B, NK, and NKT cells were seen in the spleens and lungs of healthy CB_2_^-/-^ and WT mice (**Figures S3F, G**).

**Figure 6:**
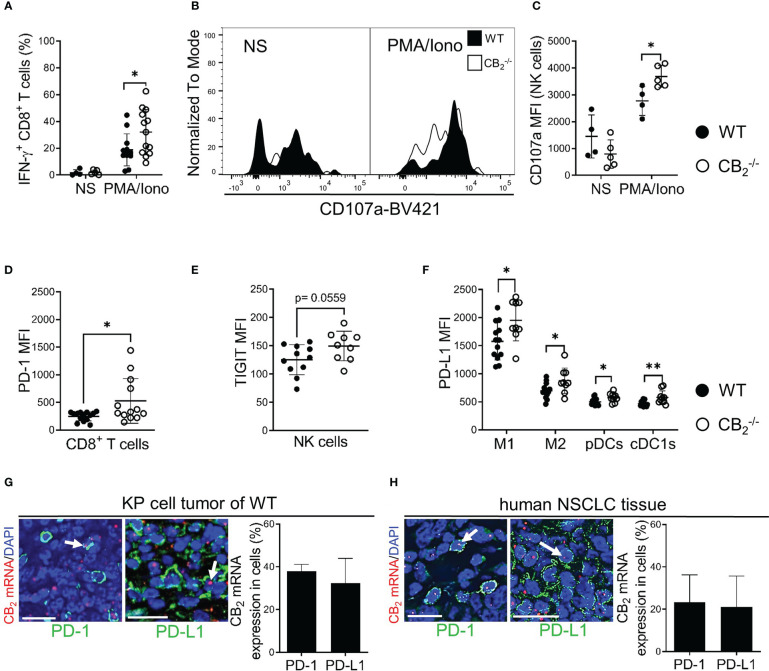
A CB_2_ deficient TME stimulates activity of CD8^+^ T and NK cells and alters expression of immune checkpoint proteins. **(A)** IFN-γ production of intratumoral CD8^+^ T (CD45^+^/CD3^+^/CD8^+^) cells prior to (non-stimulated, NS) and after *ex vivo* stimulation with phorbol myristate acetate/ionomycin (PMA/Iono). Data indicate mean values ± SD from two pooled independent experiments. n=4 (for NS); n≥11 (for PMA/Iono).**(B, C)** Degranulation capacity of tumor-infiltrated NK (CD45^+^/CD3^-^/NKp46^+^) cells before (NS) and after *ex vivo* stimulation with PMA/Iono. The graph depicts MFI of CD107a on NK cells. One representative experiment is shown. Data indicate mean values ± SD. n≥4. **(D)** MFI of PD-1 on tumor-infiltrated CD8^+^ T cells is shown. Data indicate mean values ± SD from two pooled independent experiments. n=13-14. **(E)** MFI of TIGIT on tumor-infiltrated NK cells. Data indicate mean values ± SD from two pooled independent experiments. n=13-14. **(F)** MFI of PD-L1 on tumor-infiltrated myeloid cells. Data indicate mean values ± SD from two pooled independent experiments. n=10-13. Detailed information on immune cell markers is provided in **Figure S1**. **(G)** ISH-IF analysis of KP cell tumors. Co-localization of PD-1/PD-L1 positively stained cells with CB_2_ mRNA is shown. Data indicate mean values +SD. n=3 (sections from three different tumors, 30-150 cells counted per section). **(H)** ISH-IF staining of human NSCLC tissue sections. The graph depicts co-localization of PD-1^+^/PD-L1^+^ stained cells with CB_2_ mRNA. Data indicate mean values +SD. n=3 (tumor sections from three different patients with NSCLC were used for quantification, 30-150 cells counted per section). Arrows indicate co-localization of CB_2_ mRNA with cells positive for PD-1/PD-L1. Calibration bars=20 μm. Statistical differences were evaluated by using unpaired student`s *t*-test **(D, E)**, multiple *t*-tests **(A, C, F)**. *p<.05; **p<.01. *IFN-γ*, interferon-gamma; *NK*, natural killer cells; *MFI*, median fluorescence intensity; *PD-1*, programmed death-1; *TIGIT*, T cell immunoglobulin and ITIM domain; *PD-L1*, programmed death-ligand 1; *M1*, M1 macrophages; *M2*, M2 macrophages, *pDCs*, plasmacytoid dendritic cells; *cDC1*, type 1 conventional dendritic cells, *ISH-IF*, *in situ* hybridization and immunofluorescence; *WT*, wild type; *NSCLC*, non-small cell lung cancer.

To identify underlying mechanisms of the tumor reduction in CB_2_^-/-^ mice, we checked for apoptosis and proliferation rates of tumor cells (CD45^-^) and infiltrating immune cells (CD45^+^). Flow cytometric analysis and cleaved-caspase-3/caspase-3 immunoblotting of tumors from CB_2_^-/-^ and WT mice showed no significant differences in apoptosis rates (**Figures S4B–D**). Similarly, *in vivo* and *in vitro* cell proliferation in tumor cells and infiltrating immune cells from CB_2_^-/-^ mice using bromodeoxyuridine (BrdU) incorporation assay and Ki-67 immunofluorescence did not differ from WT mice (**Figures S5B, C**). To test whether cytotoxic immune cells were more activated in the CB_2_^-/-^ mice, we stimulated tumor-infiltrating CD8^+^ T and NK cells from CB_2_^-/-^ and WT mice *ex vivo* with PMA/Iono and assessed the activity of these cells using flow cytometry. In comparison to WT mice, tumors of CB_2_^-/-^ mice showed increased expression levels of IFN-γ on CD8^+^ T cells (**Figure 6A**), and CD107a on NK cells (**Figures 6B, C**), signifying a local activation and enhanced tumoricidal activity of CD8^+^ T and NK cells. Therefore, a deficiency of CB_2_ in the TME leads to a higher number as well as to an increased activity of cytotoxic lymphocytes in the tumor.

### A CB_2_ deficient TME leads to a higher expression of immune checkpoint proteins and an enhanced responsiveness to PD-1 blocking antibodies

We next aimed to identify possible immune-based therapeutic strategies that could augment tumor reduction and hypothesized that a CB_2_ deficiency in the TME would have a favorable effect on immune checkpoint blockade. Thus, we first measured surface expression of different immune checkpoint proteins on immune cells. Results show that PD-1 expression was increased on tumor-infiltrating CD8^+^ T cells, but not on NK cells in CB_2_^-/-^ vs. WT mice. On NK cells, only TIGIT (T cell immunoglobulin and ITIM domain) showed higher expression (**Figures 6D, E**, **S6A**). We also detected enhanced expression of PD-L1 on myeloid cells (macrophages and DCs) of CB_2_^-/-^ vs. WT mice (**Figure 6F**). Regarding the other immune checkpoint proteins, no significant differences were detected for CTLA-4 (cytotoxic T-lymphocyte antigen-4), TIM-3 (T cell immunoglobulin and mucin domain-containing protein-3), and LAG-3 (lymphocyte activation gene-3) on NK and CD8^+^ T cells (**Figures S6B–H**). Dual ISH-IF revealed that approximately 40% of PD-1^+^ and PD-L1^+^ cells co-localized with CB_2_ mRNA in the KP cell tumors (**Figure 6G**). In human lung cancer, about 20% of PD-1^+^ and PD-L1^+^ cells co-localized with CB_2_ mRNA (**Figure 6H**).

**Figure 7:**
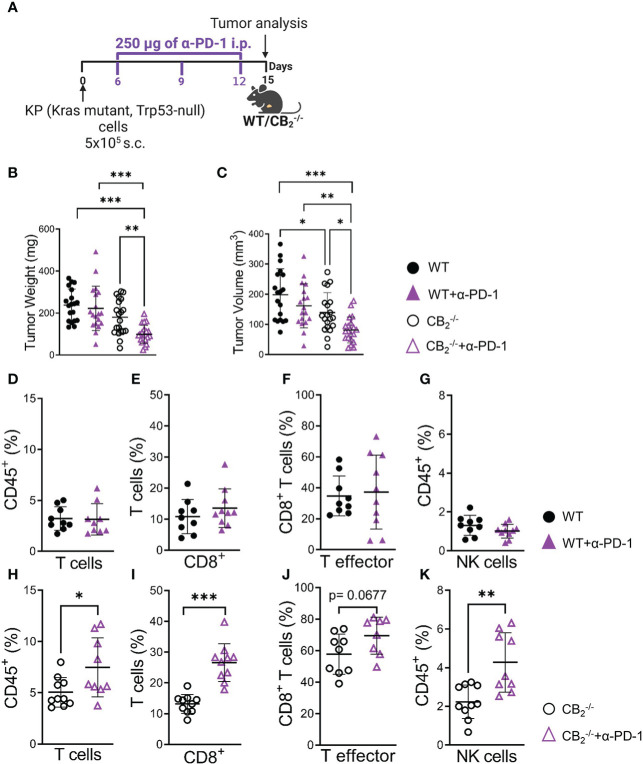
CB_2_^-/-^ mice are more responsive to anti-PD-1 antibody treatment than their wild type littermates. **(A)** Experimental design: CB_2_^-/-^ mice and WT littermates were subcutaneously (s.c.) injected with 5x10^5^ KP (Kras mutant, Trp53-null) lung adenocarcinoma cells on day 0. On days 6, 9, and 12, mice were treated with 250 µg of anti-PD-1 (α-PD-1) antibodies (or isotype control). **(B, C)** Tumor weight and volume were measured at the end of the experiment on day 15 *ex vivo*. Data indicate mean values ± SD from two pooled independent experiments. n=19-21. **(D–K)** Flow cytometric analysis was performed on single cell suspensions from KP cell tumors of CB_2_^-/-^ and WT α-PD-1 (or isotype control) treated mice. Detailed information on immune cell markers is provided in **Figure S1**. Data indicate mean values ± SD. One representative experiment is shown. n≥8. Statistical differences were evaluated by using one-way ANOVA, Tukey’s multiple comparison test **(B, C)**, unpaired student`s *t*-test **(D–K)**. *p < .05; **p < .01; ***p < .001; *WT*, wild type; *NK*, natural killer cells.

Based on these findings, we treated CB_2_^-/-^ mice with anti-PD-1 to boost immune cell activity (**Figure 7A**). Deficiency of CB_2_ on host cells augmented the responsiveness to PD-1 antibody treatment, resulting in an enhanced reduction of tumor growth in the CB_2_^-/-^ mice (**Figures 7B, C**).

Flow cytometric analysis showed that PD-1 antibody therapy potentiated an increase in the number of CD8^+^ T and NK cells in tumors of CB_2_^-/-^ mice (**Figures 7H–K**), but not in WT (**Figures 7D-G**), indicating that the deletion of CB_2_ in the TME favors an enhanced responsiveness to PD-1 therapy and causes a reduction in tumor burden.

## Discussion

For many decades, the concept that cancer development is mainly driven by genetic mutations within tumor cells, has been studied in detail. However, cancer progression is additionally regulated by the surrounding niche, called the TME, which may deliver vital factors that promote cancer development or escape from host immune surveillance (32). A number of studies have identified the significance of immune cells of the TME in tumor development and as targets in immunotherapy. As such, cytotoxic lymphocytes like CD8^+^ T and NK cells are important prerequisites for successful immunotherapy (33–37).

**Figure 8:**
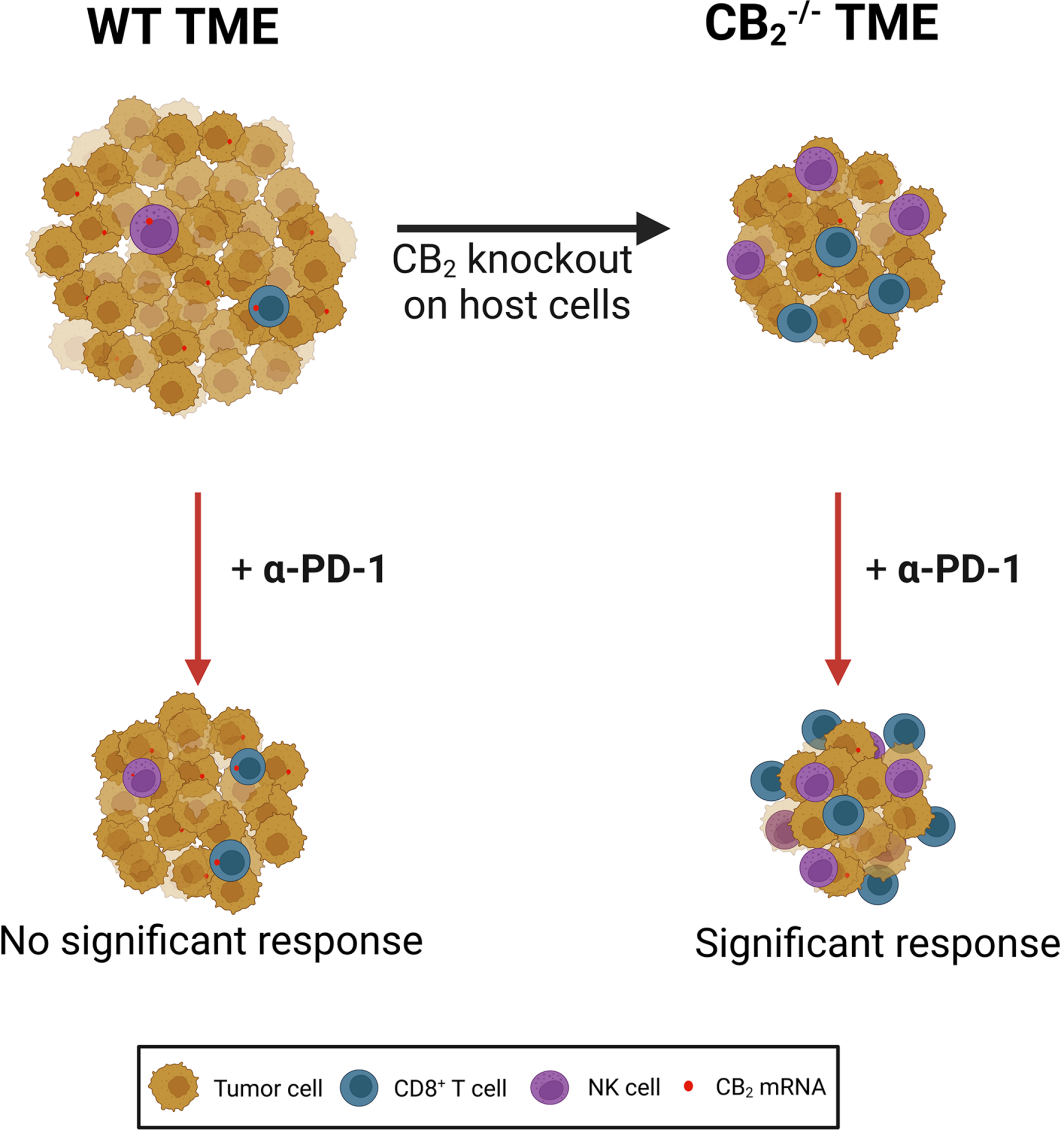
CB_2_ expressed on tumor microenvironment (TME) cells creates a pro-tumorigenic microenvironment by limiting the activity of cytotoxic lymphocytes in a mouse model of NSCLC. Deletion of the gene encoding CB_2_ on host cells results in a reduction of tumor growth as well as increased infiltration and local tumoricidal activity of CD8^+^ T and natural killer (NK) cells. CB_2_ knockout (CB_2_^-/-^) mice responded significantly better to anti-PD-1 (α-PD-1) therapy than wild type (WT) mice. α-PD-1 therapy further increased accumulation of CD8^+^ T and NK cells in the TME of CB_2_^-/-^ mice. *NSCLC*, non-small cell lung cancer.

CB_1_ and CB_2_ are over-expressed in various types of cancer, such as skin (38), breast (39) and NSCLC (4), and they have long been implicated in cancer progression (2, 3, 11, 38, 39). However, in addition to tumor cells, CB_1_ and CB_2_ are expressed in immune cells that can potentially populate the TME, where they could play a pro- or anti-tumorigenic role (27). A number of studies have focused on CB receptor/ligand interactions in tumor cells and how this axis influences tumor growth *in vitro* and *in vivo* (40), including studies in lung cancer cells and models of lung cancer (3, 4, 8). In contrast, little has been described on CB receptors in immune cells of the TME and how TME-derived CB receptors shape the immune cell profile and the response to immunotherapy. In our current study, we demonstrated that deficiency of CB_2_ in the TME host cells contributes to a reduction in tumor burden in a mouse model of NSCLC (summarized in **Figure 8**).

### CB receptors are present in tumor cells and immune cells *in situ*

Using dual ISH-IF analysis of mouse and human lung cancer sections, we revealed that tumor cells as well as tumor-infiltrating immune cells, such as CD8^+^ T, NK cells, and macrophages express CB_2_ at much higher levels than CB_1_. ISH-IF showed co-localization of CB_2_ mRNA in around 20-40% of immune cells, and 25% in KP tumor cells, suggesting TME cell-mediated and/or possible direct effects on tumor cells by CB_2_. Pharmacological activation or inhibition of CB_2_ in CB_2_^-/-^ mice (i.e., targeting only CB_2_-expressing KP tumor cells) revealed no influence of tumor cell-derived CB_2_ on tumor growth, indicating that only CB_2_ expressed in TME cells was responsible for the diminished tumor growth. The conflicting findings of CB_2_ in lung cancer (2–4), therefore, suggest a heterogeneous role for CB_2_ in lung carcinogenesis, which most likely depends not only on CB_2_ expressing tumor cells, but also on the type of TME-infiltrating immune cells expressing CB_2_.

### TME-derived CB_2_ controls immune cell infiltrates to the tumor

Cannabinoid ligands are known to suppress phagocytosis, antigen presentation, and other features of immune cells that are essential for regulation of immune activity in the TME (16). As we detected widespread CB_2_ expression in immune cells of the TME, we assessed the immune cell profile of the tumors.

Our flow cytometric analyses demonstrated that the immune cell landscape was altered in the absence of CB_2_ in the TME. Although there was no shift in the myeloid cell profile, we observed a significant infiltration of cytotoxic lymphocytes, mainly of cytotoxic CD8^+^ T and NK cells into the TME of CB_2_^-/-^ as compared to WT mice. We also found a negative correlation between the percentages of infiltrated CD8^+^ T and NK cells into the TME and the tumor weights in CB_2_ deficient mice, suggesting an involvement of CD8^+^ T and NK cells in the reduction of tumor growth. A more detailed investigation of these cells revealed that tumor-infiltrating CD8^+^ T and NK cells of CB_2_^-/-^ mice possessed higher cytotoxic activity (higher levels of IFN-γ and CD107a). These data are fully consistent with studies describing that an increased infiltration of the cytotoxic lymphocytes into the TME is associated with a good prognosis (41–43). Particularly in NSCLC, activity of CD8^+^ T and NK cells may be hampered: NK cells can overexpress inhibitory receptors (44), additionally they have been shown to poorly infiltrate NSCLC tumors, and are found more frequently in normal lung than neoplastic tissues (45). Moreover, a reduced number of cytotoxic T cells along with a reduction in IFN-γ expression was observed in NSCLC patients (46, 47). Hence, CB_2_ deficiency reversed the low infiltration of NK and CD8^+^ T cells in our model and boosted their activity, likely contributing to a reduction in tumor size.

### CB_2_^-/-^ mice are highly susceptible to PD-1 checkpoint inhibitor treatment

Immunotherapies using checkpoint inhibitors have been demonstrated to increase survival of patients in a number of cancer types, including melanoma and lung cancer (48, 49). Among all known checkpoints, the most prominent target for treatment is the PD-1/PD-L1 axis, owing to its proven efficacy in several types of cancers (48–50). Previous studies found that one of the critical requirements for ICIs to work is a sufficient infiltration of lymphocytes, including CD8^+^ T cells, at tumor sites (33, 51). A major finding of our study is that tumor-bearing CB_2_^-/-^ mice responded significantly better to anti-PD-1 treatment than the WT mice (as demonstrated by the significant reduction in tumor burden). In addition, we noticed increased PD-1 expression on CD8^+^ T cells in tumors of CB_2_^-/-^ mice, an indication of high T cell activity against tumor antigens as well as a possible prediction of anti-PD-1 therapy response (25, 34, 52). Our data also revealed increased PD-L1 expression on tumor-infiltrating myeloid cells in CB_2_^-/-^ mice, another important finding that the tumor might respond favorably to anti-PD-1 therapy (53–55). Cytotoxic CD8^+^ T cells are often the main focus of interest in terms of improving immune checkpoint blockade therapies, but other immune cells, such as NK cells may provide an important contribution to the efficacy of checkpoint inhibitors (reviewed in (56)). Thus, the presence of intratumoral cytotoxic NK cells promotes a positive response to immunotherapies, by also targeting the PD-1/PD-L1 axis (35, 36). Recent studies found that the number of NK cells correlated with the responsiveness to anti-PD-1 treatment, and improved overall survival in melanoma and metastatic melanoma patients (37, 43). Zhang et al. identified that the presence of NK cells provided an enhanced clinical benefit of PD-L1 as well as TIGIT-based immunotherapies, as NK cells improved the functional role of CD8^+^ T cells and/or inhibited their exhaustion (57). The TME of CB_2_^-/-^ mice had a significantly higher number of NK cells than WTs, and their presence, therefore, may enhance the susceptibility to immunotherapy with anti-PD-1.

To further assess susceptibility to checkpoint blockade, we determined other proteins that inhibit T and NK cells activity/proliferation, such as CTLA-4, TIM-3, TIGIT, and LAG-3 (58–62). Except for increased expression of TIGIT on NK cells, there were no significant differences between CB_2_^-/-^ and WT mice littermates as to the expression rates of these proteins on CD8^+^ T and NK cells. Collectively, our data suggest that CD8^+^ T and NK cells in CB_2_^-/-^ mice were in an active, non-exhausted state (high levels of IFN-γ and PD-1 on CD8^+^ T cells, and of CD107a on NK cells).

### Deficiency of CB_2_ in the TME increases the PD-1 antibody-induced effect on CD8^+^ T and NK cells

The effect of an anti-PD-1/PD-L1 therapy on the immune cell composition has often been associated with the restoration of effector CD8^+^ T cell activity to kill tumor cells (63). Other cytotoxic lymphocytes, including NK cells, also contribute to the response to immunotherapy (reviewed in (64)): Lee et al. demonstrated increased frequency of intratumoral and peritumoral NK cells in melanoma patients who responded well to PD-1 blockade (37). Hsu et al. also identified that, in addition to T cells, NK cells can mediate the effect of anti-PD-1/PD-L1 therapy (35). In our study, the anti-PD-1 therapy further increased the number of CD8^+^ T and NK cells at the tumor site of CB_2_^-/-^ as compared to WT mice. This supports the concept that a successful anti-PD-1 therapy is inherently linked to the presence of CD8^+^ T and NK cells in the TME. It should be mentioned that PD-1 expression in tumor-infiltrating NK cells of CB_2_^-/-^ mice was not different from WT mice, and that PD-1 expression was lower on NK than CD8^+^ T cells. This calls into question whether there is a direct effect of anti-PD-1 antibodies on NK cells, as the checkpoint blockade may have indirectly modulated anti-cancer NK cell functions *via* the crosstalk with other immune cell populations, as previously described (65, 66). While this manuscript was in preparation, a study was published, describing that THC and exogenous cannabinoids (approved for the treatment of chemotherapy-induced nausea) reduced the effect of anti-PD-1 therapy (67), reconfirming our own observations. Cannabis is well-known for its immunosuppressive effects (68), which is also supported by a recent observation that the use of cannabis during treatment with PD-1 checkpoint inhibitor nivolumab in cancer patients lowered their response rate (69). With our study, we highlight a possible mechanism for a lower response, which includes CB_2_, CD8^+^ T and NK cells.

## Conclusion

Our results demonstrate that the CB_2_ receptor in the TME of NSCLC tumors may act as an immunosuppressor that impedes CD8^+^ T and NK cell activity, thereby promoting tumor growth. Deletion of CB_2_ in the TME releases the immunosuppressive break rendering tumors to be more susceptible to PD-1 inhibitor treatment. The findings also suggest that the use of cannabis or cannabinoid-based medicine during immunotherapy may lead to a low treatment response. Altogether, the CB_2_ receptor maybe an interesting adjuvant target for ICI therapy.

## Materials and methods

### Cancer cell lines and mice

The mouse KP cell line (a generous gift by Dr. McGarry Houghton from the Fred Hutchinson Cancer Center, Seattle, USA) was isolated from a lung adenocarcinoma, grown in a Kras mutant/Trp53-null (Kras^LSL-G12D^/p53^fl/fl^) mouse following intratracheal administration of adenoviral Cre recombinase, as described before (70). Briefly, pieces of mechanically disintegrated lung tumor were cultured in Dulbecco’s Modified Eagle Medium (DMEM) supplemented with FBS (10%), penicillin (100units/mL) and streptomycin (100μg/mL). Clonal cells were derived by single-cell dilution into 96 well plates (70). Lewis lung carcinoma (LLC1) cell line was purchased from ATCC (Rockville, Maryland, USA). Both cell lines were maintained in DMEM media containing 10% fetal bovine serum (FBS, Life Technologies) and 1% penicillin/streptomycin (P/S, PAA Laboratories) and kept in a humidified incubator (5% CO_2_) at 37°C and passaged every 48 hrs. The cell lines were mycoplasma free.

All animals were bred and maintained in the animal facilities of the Medical University of Graz. Wild type C57BL/6J (B6) mice were purchased from Charles River, Germany. CB_1_^-/-^ mice on B6 background were obtained from Dr. Andreas Zimmer, University of Bonn, Germany. CB_2_^-/-^ mice (B6.129P2-Cnr2^tm1Dgen^/J on B6 background) were obtained from Jackson Laboratories (Bar Harbor, ME, USA). Experiments were performed on 6-14-week-old mice. All procedures were granted by the Austrian Federal Ministry of Science and Research (protocol number: BMBWF-66.010/0041-V/3b/2018). Subcutaneous (s.c.) injections of KP or LLC1 cells were performed under inhaled isoflurane anaesthesia. To generate s.c. tumors, KP or LLC1 cells (5×10^5^) suspended in 450 µL Dulbecco’s Phosphate Buffered Saline (PBS, Gibco) were injected s.c. into the lower flanks of mice on day 0. Tumors were harvested at the experimental endpoint (day 15 for KP cell tumor model; day 21 for LLC1 lung tumor model) and were subsequently weighed, measured with a digital caliper *ex vivo*, and collected for analysis. Tumor volume was calculated based on the following formula: v = length x width x height x π/6 (71).

### Pharmacology

To pharmacologically block CB_1_ receptors, tumor-bearing C57BL/6J WT mice were intraperitoneally (i.p.) treated with 1 mg/kg/d SR141716 (28, 29) (CB_1_ antagonist, Cayman Chemical, Ann Arbor, MI). For pharmacological activation of CB_2_ receptors, tumor-bearing CB_2_^-/-^ mice were i.p. treated with 20 mg/kg/d JWH-133 (31) (CB_2_ agonist, Axon Medchem, Groningen, NL). To pharmacologically block CB_2_ receptors, tumor-bearing CB_2_^-/-^ mice and C57BL/6J WT mice were i.p. treated with 10 mg/kg/d SR144528 (29, 30) (CB_2_ antagonist, Cayman Chemical, Ann Arbor, MI) or vehicle (ethanol). The treatment period for all mentioned interventions was ten days, starting from day 5 when the s.c. tumors were palpable, until day 14. For inhibition of PD-1, tumor-bearing CB_2_^-/-^ mice and WT littermates were injected i.p. with 250 μg of rat monoclonal anti-mouse PD-1 antibody (72) (clone 29F.1A12, BioXCell, Lebanon, NH) or rat IgG2a isotype control (clone 2A3, BioXCell, Lebanon, NH) on days 6, 9, and 12.

### Single-cell suspensions

Single cell suspensions of dissected mouse KP cell tumors were prepared as previously described (71). Briefly, using surgical scissors, tumors were cut into small pieces, and afterwards digested with DNase I (160 U/ml; Worthington) and collagenase (4.5 U/ml; Worthington) for 20 min at 37°C, while rotating at 800-1000 rpm. The tissue was then passed through a 40 μm cell strainer, washed in staining buffer (SB, PBS+2% FBS), suspended in PBS, counted, and used for surface, intracellular and nuclear antigen staining.

### Flow cytometry of dissected KP cell tumors

To exclude dead cells, single cell suspensions were initially incubated for 20 min in Fixable Viability Dye (FVD) eFluor™ 780 (eBioscience) at 4°C in the dark. Prior to staining with surface, intracellular and nuclear antibodies, single cell suspensions were incubated in 1 μg TruStain FcX™ (BioLegend, # 101320) for 10 min at 4°C. Immunostaining was performed for 30 min at 4°C (protected from light) using the pre-mixed antibody panels (**Table S1**). To detect FoxP3 nuclear antigen within the cells, surface stained cells were permeabilized and fixed with Transcription Factor Buffer Set (BD Biosciences, # 562574) before staining with FoxP3 antibody (**Table S1**). To detect expression of IFN-γ and CD107a, single-cell suspensions of the tumors (2×10^6^ cells per well) were suspended in RPMI media supplemented with 10% FBS, 1% P/S, and GolgiStop (1.5 μl/ml, BD Biosciences), seeded into 96- well U-bottomed plates, and incubated for 4 hrs at 37°C (5% CO_2_). During incubation time, CD107a was added, and cells were stimulated with phorbol myristate acetate (PMA) (100 ng/ml, Sigma Aldrich) and ionomycin (Iono) (1 μg/ml, Sigma Aldrich), or used unstimulated (73, 74). Afterwards, surface and intracellular stainings (BD Cytofix/Cytoperm™ Kit) were performed with the pre-mixed antibody panel (**Table S1**). Cells were then washed and fixed in eBioscience™ IC Fixation Buffer (ThermoFisher Scientific, # 00-8222-49) for 10 min at 4°C. Fixed cells were either acquired on a BD LSR Fortessa™ or a BD Canto™ flow cytometer connected to FACSDiva software (BD Biosciences). FlowJo software (Treestar) was used for analysis and compensation. Fluorescence minus-one-samples were used to define gates (**Figures S1A-D**).

### RNA extraction and RT-qPCR

RNA was extracted from tissue and KP cells using Trizol (Life Technologies) and RNeasy Kit (Qiagen), respectively. Samples were treated either with a DNA-*free*™ DNA Removal Kit (Invitrogen) or RNase-Free DNase set (Qiagen). The quality and concentration of RNA were determined using a NanoDrop ND-1000 spectrophotometer (Thermo Fisher Scientific). Reverse transcription of purified RNA (1 µg) was performed by High-Capacity cDNA Reverse Transcription Kit (Applied Biosystems). Gene expression was assessed by reverse transcription-quantitative polymerase chain reaction (RT-qPCR) using SsoAdvanced Universal SYBR Green Supermix (Bio-Rad). Primers were acquired from Eurofins (**Table S2**) and Bio-Rad (**Table S3**). Relative gene expression was calculated using 2^-ΔΔCT^ methods (75).

### *In situ* hybridization and immunofluorescence

#### Mouse and human NSCLC tissue samples

Tumors from mice were fixed in acid-free phosphate-buffered 10% formaldehyde solution (Roti^®^- Histofix 10%, pH 7) for 24-48 hrs at room temperature with gentle shaking. Tissue was further processed for paraffin embedding based on standard procedures. Human NSCLC tissue samples (formalin-fixed and paraffin-embedded) were acquired from the Biobank of the Medical University of Graz. Ethical approval was obtained from the Institutional Review Board of the Medical University of Graz (EK-numbers: 30-105 ex 17/18). All procedures involving clinical samples followed the ethical standards of the institutional and/or national research committee and the 1964 Helsinki Declaration and its later amendments or comparable ethical standards. All patients participated in the study gave informed consent.

ISH probes used to detect CB_1_ and CB_2_ mRNAs in mouse tumor and human NSCLC tissue were purchased from Advanced Cell Diagnostics (ACD, Newark, USA) (**Table S4**). ISH was performed using RNAscope^®^ 2.5 HD red kit according to manufacturer’s instructions. Briefly, tumor tissue sections were first treated with H_2_O_2_ at room temperature for 10 min and target retrieval was performed using the Brown FS3000 food steamer at 95°C for 15 min. Then, the sections were digested with protease IV in HybEZ™ II oven (ACD, Newark, USA) at 40°C for 20 min, washed in distilled water, followed by incubation with the corresponding probes at 40°C for 2 hrs and stained with Fast Red. To compare tissue samples from CB_1_^-/-^ or CB_2_^-/-^ and WT mice, sections were placed on a single slide. The specificity of the mouse CB_1_ and CB_2_ probes was previously verified in CB_1_^-/-^ and CB_2_^-/-^ mice (76). Immunofluorescence of tumor cells and infiltrated immune cells of the TME was conducted using primary antibodies listed in **Table S5**. Alexa Fluor^®^ 488-labeled goat anti-rabbit IgG (1:500, Jackson Immuno Research, #111-546-144) and Alexa Fluor^®^ 488-labelled bovine anti-goat IgG (H+L) (1:500, Jackson Immuno Research, # 805-545-180) were used as secondary antibodies. In parallel, sections were processed in the absence of primary antibody as a negative control. Then, sections were mounted with Vectashield^®^ (containing DAPI) (Vector Laboratories) and images were taken using an Olympus IX73 fluorescence microscope (Olympus) connected with a Hamamatsu ORCA-ER digital camera (Hamamatsu Photonics K.K., Japan). Images were processed with an Olympus CellSens^®^ 1.17 imaging software containing a deconvolution program (Olympus). ImageJ software was used to quantify expression and co-localization with the corresponding probes.

### Statistical analysis

Data are presented as means ± standard deviation (SD) or standard error of means (SEM) and analyzed using Prism v.9.3.1 (GraphPad Software, La Jolla, CA, USA). Differences between experimental groups were assessed by unpaired student’s t-tests, multiple t-tests or two-way analysis of variance (ANOVA) with the indicated *post hoc* test for corrections of multiple comparisons, whereas for multiple comparisons with three or more experimental groups, a one-way ANOVA was applied with the indicated *post hoc* test for corrections of multiple comparisons. Shapiro-Wilk and Kolmogorov-Smirnov tests were used to test a normal distribution. Correlations between tumor weight and infiltration of CD8^+^ T and NK cells in the TME was determined using Pearson’s correlation coefficient (r_p_) and Spearman’s correlation coefficient *rho* (r_s_).

In all cases, a p-value <0.05 was considered significant and represented with one, two or three asterisks when lower than 0.05, 0.01, or 0.001, respectively.

## Data availability statement

The original contributions presented in the study are included in the article/**Supplementary Material**. Further inquiries can be directed to the corresponding author.

## Ethics statement

The studies involving human participants were reviewed and approved by the Ethics Committee of the Medical University of Graz. The patients/participants provided their written informed consent to participate in this study. The animal study was reviewed and approved by the Austrian Federal Ministry of Education, Science and Research.

## Author contributions

ArS, MK, JK, CH and RS contributed to the conception and design of the study. ArS, MK, EG, DR, CH, KM, AnS and PVC performed experiments and acquired data. ArS, MK, EG, JK and RS contributed to the analysis and interpretation of the data. ArS and RS participated in the writing of the manuscript. LB and JL provided the human lung cancer samples. All authors contributed to the article and approved the submitted version.

## Glossary

**Table:**

ANOVA	Analysis of variance
ATR	Ataxia telangiectasia and Rad3-related
BrdU	Bromodeoxyuridine
CB	Cannabinoid
CB_1_^-/-^	CB_1_ knockout
CB_2_^-/-^	CB_2_ knockout
cDC1	Type 1 conventional dendritic cells
CK	Cytokeratin
CTLA-4	Cytotoxic T-lymphocyte antigen-4
DC	Dendritic cell
DMEM	Dulbecco’s Modified Eagle Medium
ECS	Endocannabinoid system
EGF	Epidermal growth factor
EPCAM	Epithelial cell adhesion molecule
FBS	Fetal bovine serum
FVD	Fixable Viability Dye
i.p.	Intraperitoneal
ICI	Immune checkpoint inhibitor
IFN-γ	Interferon-gamma
IL-10	Interleukin-10
IL-2	Interleukin-2
ISH-IF	In situ hybridization and immunofluorescence
LAG-3	Lymphocyte activation gene-3
LLC1	Lewis lung carcinoma
M1	M1 macrophages
M2	M2 macrophages
MEK	Mitogen-activated protein kinase kinase
MFI	Median fluorescence intensity
NK	Natural killer cells
NKT	Natural killer T cells
NS	Non-stimulated
NSCLC	Non-small cell lung cancer
P/S	Penicillin/streptomycin
PBS	Phosphate Buffered Saline
PD-1	Programmed death-1
pDC	Plasmacytoid dendritic cells
PD-L1	Programmed death-ligand 1
PMA/Iono	Phorbol myristate acetate/Ionomycin
PVDF	Polyvinylidene difluoride
RBC	Red blood cell
RT-qPCR	Reverse transcription-quantitative polymerase chain reaction
s.c.	Subcutaneous
SB	Staining buffer
SD	Standard deviation
SEM	Standard error of mean
TBST	Tris-buffered saline with 0.1% Tween® 20 Detergent
TGF	Transforming growth factor
Th_1_	T helper 1
Th_2_	T helper 2
THC	Tetrahydrocannabinol
TIGIT	T cell immunoglobulin and ITIM domain
TIM-3	T cell immunoglobulin and mucin domain-containing protein-3
TME	Tumor microenvironment
TNF-α	Tumor necrosis factor-alpha
Tregs	Regulatory T
WB	Western blotting
WT	Wild type
