# Supplementary material for: Cannabinoid receptor 2 plays a pro-tumorigenic role in non-small cell lung cancer by limiting anti-tumor activity of CD8+ T and NK cells
Source: Front Immunol. 2023 Jan 9;13:997115. doi: 10.3389/fimmu.2022.997115 (PMC9868666; doi:10.3389/fimmu.2022.997115)

**SUPPLEMENTAL MATERIAL**

**Table of Contents**
Supplemental Material and Methods

Reference list
Tables S1-5
Figures S1-6

**SUPPLEMENTAL MATERIAL AND METHODS**

**Flow Cytometry of Human NSCLC Tissues**

*Study design and approval*

Patients with NSCLC stages IA-IIIB were recruited from the Department of Internal Medicine, Department of Oncology and Department of Surgery, Division of Thoracic Surgery, and Medical University of Graz (Graz, Austria). Informed consent was obtained from all participants. The study complied with the Declaration of Helsinki and was approved by the *Ethics Committee of the Medical University of Graz* (protocol number: 30-105 ex17/18).

*Single cell suspension*

Single cell suspensions of lung tumors were prepared as previously described (1). Briefly, tissues were cut into small pieces, and digested in RPMI media containing DNase I (40 U/ml; Worthington) and collagenase (150 U/ml; Worthington) for 30 min at 37°C. Afterwards, tissue was passed through a 100 μm cell strainer, suspended in staining buffer (SB, PBS supplemented with 2% FBS), and centrifuged for 5 min at 500 g (4°C). The pellet was resuspended in RBC lysis buffer (BioLegend, # 420301) and incubated for 3 min on ice with occasional shaking. Lysis process was neutralized by adding four volumes of PBS. Then, cells were passed through a 40 μm cell strainer onto a fresh 50 ml tube and washed in SB. Subsequently, cells were washed, resuspended in PBS, counted, and then used for antigen staining.

*Flow cytometry*

Cells (2x10^6^ per 96-well plate) were first incubated for 20 min in Fixable Viability Dye (FVD) eFluor^TM^ 780 (eBioscience) in the dark at 4°C to exclude dead cells, and then stained with anti-CB_2_ antibody (1:50, Abcam, # ab3561) for 45 min at 4°C. Afterwards, cells were stained with a goat anti-rabbit IgG H&L (Alexa Fuor® 488) secondary antibody (1:500, Abcam, # ab150077) for 45 min at 4°C followed by pre-incubation with human TruStain FcX^TM^ (Fc Receptor Blocking Solution, BioLegend, # 422302) for 10 min at 4°C, and further stained with a pre-mixed panel of antibodies for 20 min at 4°C (**Table S1**). Thereafter, cells were washed and fixed in eBioscience^TM^ IC Fixation Buffer (ThermoFisher Scientific, # 00-8222-49) for 10 min at 4°C. Fixed cells were measured on a BD LSR Fortessa^TM^ flow cytometer equipped with FACSDiva software (BD Biosciences). FlowJo software (Treestar) was used for data analysis and compensation. Fluorescence minus-one-samples were used to define gates (**Figure S1E**).

**Flow Cytometry of mice tissues**

*Single cell suspension from spleen and lung*

Spleen and lung tissues were extracted from healthy age-matched CB_2_ knockout (CB_2_^-/-^) and wild type (WT) littermates and subsequently processed for flow cytometry analysis.

Briefly, whole spleens were minced with a syringe plunger and passed through a 40 µm cell strainer, suspended in SB, and centrifuged at 4°C for 5 min at 500 g. Then, the supernatant was discarded and the pellet was resuspended in 5 mL of RBC lysis buffer (BioLegend, # 420301) and incubated for 5 min on ice with occasional shaking. To stop lysis process, four volumes of PBS were added and centrifuged at 4°C for 5 min at 500 g. Then, cells were washed two times in PBS, resuspended in PBS, counted, and then used for surface antigen staining.

Single cell suspensions of lung tissues were prepared as previously described (1). Briefly, tissues were cut into small pieces, and digested in RPMI media containing DNase I (40 U/ml; Worthington) and collagenase (150 U/ml; Worthington) for 30 min at 37°C. After incubation, tissue was passed through a 40 μm cell strainer, suspended in SB, and centrifuged for 5 min at 500 g (4°C). The pellet was then incubated in 5 mL of RBC lysis buffer (BioLegend, # 420301) for 5 min on ice with occasional shaking. To terminate the process of lysis, four volumes of PBS were added and centrifuged at 4°C for 5 min at 500 g. Then, cells were washed two times in PBS, resuspended in PBS, counted, and then used for surface antigen staining.

*Flow cytometry*

To exclude dead cells, single cell suspensions were initially incubated for 20 min in Fixable Viability Dye (FVD) eFluor^TM^ 780 (eBioscience) at 4°C in the dark. Prior to staining with surface antibodies, single cell suspensions were incubated in 1 μg TruStain FcX^TM^ (BioLegend, # 101320) for 10 min at 4°C. Immunostaining was performed for 30 min at 4°C (protected from light) using a pre-mixed antibody panel (**Table S1**). Cells were then washed and fixed in eBioscience^TM^ IC Fixation Buffer (ThermoFisher Scientific, # 00-8222-49) for 10 min at 4°C. Fixed cells were measured on a BD LSR Fortessa^TM^ flow cytometer equipped with FACSDiva software (BD Biosciences). FlowJo software (Treestar) was used for analysis and compensation. Fluorescence minus-one-samples were used to define gates (**Figure S1A**).

**Determination Apoptosis of Tumor and Tumor-Infiltrated Immune Cells**

CB_2_ knockout (CB_2_^-/-^) mice and WT littermates were subcutaneously (s.c.) injected with 5x10^5^ KP cells on day 0. Tumors were harvested at the experimental endpoint (day 15), and were subsequently weighted, measured with a digital caliper *ex vivo*, and collected for apoptosis analysis. Afterwards, tumors were processed for flow cytometry. Briefly, antigen staining was performed for 30 min at 4°C with a pre-mixed antibody panel (**Table S1**). Then, cells were assessed for apoptosis using FITC Annexin V Apoptosis Detection Kit (BD Biosciences, # 556547). Stained cells were acquired on a BD Canto^TM^ flow cytometer with FACSDiva software (BD Biosciences). FlowJo software (Treestar) was used for analysis and compensation. Fluorescence minus-one-samples were used to define gates (**Figure S1F**).

**Western Blotting**

The level of caspase protein in KP cell tumors was determined by Western Blot (WB) analysis, where 30 μg of protein was separated using 5-12% Bis Tris gradient gel and transferred to polyvinylidene difluoride (PVDF) membranes. β-actin was used as an internal loading control on the stripped blot to confirm that protein loading was the same across all samples. Blocking was conducted at room temperature for 1 hr using 5% skimmed dry milk in tris-buffered saline with 0.1% Tween® 20 Detergent (TBST) buffer. Primary antibodies against caspase-3 (1:1000, Cell Signaling Technology, # 9662; antibody detects full-length caspase-3 (35 kDa) and the large fragment of caspase-3 resulting from cleavage (17 kDa)) and β-actin (1:5000, Sigma, # A5316, 42kDa) were incubated overnight at 4°C under gentle shaking. After extensive washes (four times for 15 min each) with a wash buffer, incubation with a horseradish peroxidase conjugated secondary antibody (goat anti-rabbit, 1:5000, Jackson ImmunoResearch, # 111-035-045) for 1 hr at room temperature was performed. Proteins were visualized using Clarity^TM^ Western ECL substrate (Bio-Rad, # 170-5061). Images of the membrane were developed with a ChemiDoc^TM^ MP Imaging System. Density of bands were quantified using the Image Lab 5.2 software from Bio-Rad. Caspase-3/cleaved caspase-3 bands were first normalized to β-actin and then a ratio was calculated (2).

***In Vivo* Assessment of Proliferation of Tumor Cells and Infiltrating Immune Cells**

CB_2_^-/-^ mice and WT littermates were s.c. injected with 5x10^5^ KP cells on day 0. On day 14, mice were injected intraperitoneally (i.p.) with 1.5 mg/kg of bromodeoxyuridine (BrdU) solution to assess proliferation within the KP cell tumor. On the following day, tumors were harvested for proliferation analysis. Afterwards, the tumors were processed for flow cytometry. Briefly, antigen staining was performed for 30 min at 4°C (protected from light) with a pre-mixed antibody panel (**Table S1**). Stained cells were acquired on a BD Canto^TM^ flow cytometer with FACSDiva software (BD Biosciences). FlowJo software (Treestar) was used for analysis and compensation. Fluorescence minus-one-samples were used to define gates (**Figure S1G**).

**Immunofluorescence for Ki-67**

Collected KP cell tumors were fixed in acid-free phosphate-buffered 10% formaldehyde solution (Roti^®^- Histofix 10%, pH 7) for 24-48 hrs at room temperature by gentle shaking, and further processed for paraffin embedding based on standard procedures. Tissue was then cut in 5 μm sections, baked at 60°C for 1 hr, de-waxed, and rehydrated. Antigen retrieval was performed by boiling the sections in sodium citrate in a microwave for 10 min. Endogenous peroxidase activity was abrogated by treatment with 0.3% hydrogen peroxide in PBS at room temperature for 30 min. A blocking step with 0.1 M PBS, containing 0.3 % Triton X-100, 1 % goat serum was performed at room temperature for 3 hrs. The primary antibody was applied overnight at 4°C (**Table S5**). Alexa Fluor® 488-labeled goat anti-rabbit IgG (1:500, Jackson Immuno Research, #111-546-144) was used as a secondary antibody. Then, sections were mounted with Vectashield® (containing DAPI) (Vector Laboratories) and images were taken by an Olympus IX73 fluorescence microscope (Olympus) connected with a Hamamatsu ORCA-ER digital camera (Hamamatsu Photonics K.K., Japan). Images were processed with an Olympus CellSens® 1.17 imaging software (Olympus). ImageJ software was used to quantify Ki-67 positive cells.

**REFERENCE LIST**

1. Kienzl M, Hasenoehrl C, Valadez-Cosmes P, Maitz K, Sarsembayeva A, Sturm E, et al. IL-33 reduces tumor growth in models of colorectal cancer with the help of eosinophils. *Oncoimmunology* (2020) 9:1-12. doi:10.1080/2162402X.2020.1776059

2. Wei M, Guo M, Meng X, Li L, Wang H, Zhang M, et al. PPARγ Mediates the Cardioprotective Roles of Danlou Tablet After Acute Myocardial Ischemia-Reperfusion Injury. *Front Cardiovasc Med* (2022) 9:858909. doi:10.3389/fcvm.2022.858909

**Tables S1-5**

**Table S1. Mouse and human flow cytometry antibody panels**

| Panel | Antibody | Dilution | Clone | Company | Catalogue # |
| --- | --- | --- | --- | --- | --- |
| Tumor-infiltrating lymphoid immune cells | CD45-AF700 | 1:200 | 30-F11 | BioLegend | 103128 |
|  | CD3-BUV395 | 1:40 | 145-2C11 | BD Biosciences | 563565 |
|  | CD8-PerCPCy5.5 | 1:80 | 53-6.7 | BioLegend | 100734 |
|  | CD4-BUV496 | 1:80 | GK1.5 | BD Biosciences | 564667 |
|  | PD-1-APC | 1:40 | 29F.1A12 | BioLegend | 135210 |
|  | CD62L-BV605 | 1:50 | MEL-14 | BioLegend | 104438 |
|  | CD44-BUV737 | 1:160 | IM7 | BD Biosciences | 612799 |
|  | NKp46-BV510 | 1:20 | 29A1.4 | BioLegend | 137623 |
|  | CD19-FITC | 1:160 | 6D5 | BioLegend | 115506 |
|  | FoxP3-PE | 1:40 | FJK-16s | eBioscience^TM^ | 12-5773-82 |
| Tumor- infiltrating myeloid immune cells | CD45-BV785 | 1:160 | 30-F11 | BioLegend | 103149 |
|  | Ly6C-APC | 1:79 | HK1.4 | BioLegend | 128015 |
|  | Ly6G-PE /Dazzle | 1:166 | 1A8 | BioLegend | 127648 |
|  | CD11c-BV605 | 1:20 | N418 | BioLegend | 117334 |
|  | PD-L1-PeCy7 | 1:79 | 10F.9G2 | BioLegend | 124313 |
|  | CD206-FITC | 1:160 | C068C2 | BioLegend | 141703 |
|  | MHCII-PerCP-Cy5.5 | 1:160 | M5/114.15.2 | BioLegend | 107625 |
|  | CD103-BV510 | 1:40 | 2E7 | BioLegend | 121423 |
|  | CD11b-BUV737 | 1:80 | M1/70 | BD Biosciences | 612801 |
|  | F4/80-BUV395 | 1:40 | T45-2342 | BD Biosciences | 565614 |
|  | Siglec-F-PE | 1:40 | E50-2440 | BD Biosciences | 562068 |
| IFN-γ and CD107a expression | CD107a-BV421 | 1:100 | 1D4B | BioLegend | 121618 |
|  | CD45-AF700 | 1:200 | 30-F11 | BioLegend | 103128 |
|  | CD3-BUV395 | 1:40 | 145-2C11 | BD Biosciences | 563565 |
|  | CD8-PerCP-Cy5.5 | 1:80 | 53-6.7 | BioLegend | 100734 |
|  | NKp46-BV510 | 1:20 | 29A1.4 | BioLegend | 137623 |
|  | IFN-γ-PE-CF594 | 1:25 | XMG1.2 | BD Biosciences | 562303 |
| Immune checkpoint protein expression | CD45-AF700 | 1:200 | 30-F11 | BioLegend | 103128 |
|  | CD3-BUV395 | 1:40 | 145-2C11 | BD Biosciences | 563565 |
|  | CD8-PerCP-Cy5.5 | 1:80 | 53-6.7 | BioLegend | 100734 |
|  | NKp46-BV510 | 1:20 | 29A1.4 | BioLegend | 137623 |
|  | CD19-PECy7 | 1:161 | 6D5 | BioLegend | 115520 |
|  | TIGIT-BV421 | 1:33 | 1G9 | BD Biosciences | 565270 |
|  | TIM-3-BV785 | 1:40 | RMT3-23 | BioLegend | 119725 |
|  | LAG-3-BV650 | 1:161 | C9B7W | BioLegend | 125227 |
|  | CTLA-4-PE | 1:20 | UC10-4B9 | BioLegend | 106305 |
| CB_2_ expression in human NSCLC | CD45-AF700 | 1:100 | HI30 | BioLegend | 304024 |
|  | CD3-APC | 1:40 | UCHT1 | BioLegend | 300411 |
|  | CD8-BV650 | 1:50 | RPA-T8 | BioLegend | 301041 |
|  | CD56-PE | 1:80 | HCD56 | BioLegend | 318306 |
|  | Epcam-PE/Dazzle | 1:17 | 9C4 | BioLegend | 324231 |
|  | CD4-BUV395 | 1:20 | SK3 | BD Biosciences | 563550 |
| Apoptosis of tumor cells and tumor-infiltrating immune cells | CD45-APC | 1:100 | 30-F11 | BioLegend | 103112 |
| Proliferation of tumor cells and tumor infiltrating immune Cells | CD45-AF700 | 1:200 | 30-F11 | BioLegend | 103128 |
|  | BrdU-FITC | 1:50 | 3D4 | BioLegend | 364104 |

**Table S2. Eurofins primer used for RT-qPCR**

| Target | Species | Sequence | Direction |
| --- | --- | --- | --- |
| *Cnr2* PrimerBank ID: 31981837a1 | mouse | ACGGTGGCTTGGAGTTCAAC | forward |
|  |  | GCCGGGAGGACAGGATAAT | reverse |

**Table S3. Bio-Rad primer used for RT-qPCR**

| Target | Species | ID |
| --- | --- | --- |
| *Hprt* | mouse | qMmuCED0045738 |

**Table S4. 20 ZZ probes used for in situ hybridization**

| Probe | Targeting bases |
| --- | --- |
| Murine CB_1_ | 701-1792 of NM_007726.3 |
| Murine CB_2_ | 291-719 of NM_009924.3 |
| Human CB_1_ | 2504-3609 of NM_033181.3 |
| Human CB_2_ | 141-1193 of NM_001841.2 |

**Table S5. List of primary antibodies used in immunofluorescence**

| Antibody | Species | Dilution/ Concentration | Company | Catalogue # |
| --- | --- | --- | --- | --- |
| Cytokeratin | Rabbit | 1:200 | Dako | ZO0622 |
| CD3 | Rabbit | 1:200 | Novus Biologicals | NB600-1441 |
| CD8 | Rabbit | 1:100 | Abcam | ab203035 |
| Mouse NKp46/NCR1 | Goat | 10µg/mL | R&D systems | AF2225 |
| CD163 | Rabbit | 1:200 | Abcam | ab182422 |
| F4/80 | Rabbit | 1:500 | Cell Signaling Technology | 70076 |
| CD11b | Rabbit | 1:200 | Novus Biologicals | NB110-89474SS |
| Human NKp46/NCR1 | Goat | 15µg/mL | R&D systems | AF1850 |
| Mouse PD-L1 | Goat | 7µg/mL | R&D systems | AF1019 |
| Mouse PD-1 | Rabbit | 1:250 | Cell Signaling Technology | D7D5W |
| Human PD-L1 | Goat | 15 µg/mL | R&D systems | MAB1561 |
| Human PD-1 | Rabbit | 1:250 | Cell Signaling Technology | D4W2J |
| Ki-67 | Rabbit | 1:400 | Cell Signaling Technology | 12202 |

**Figures S1-6**


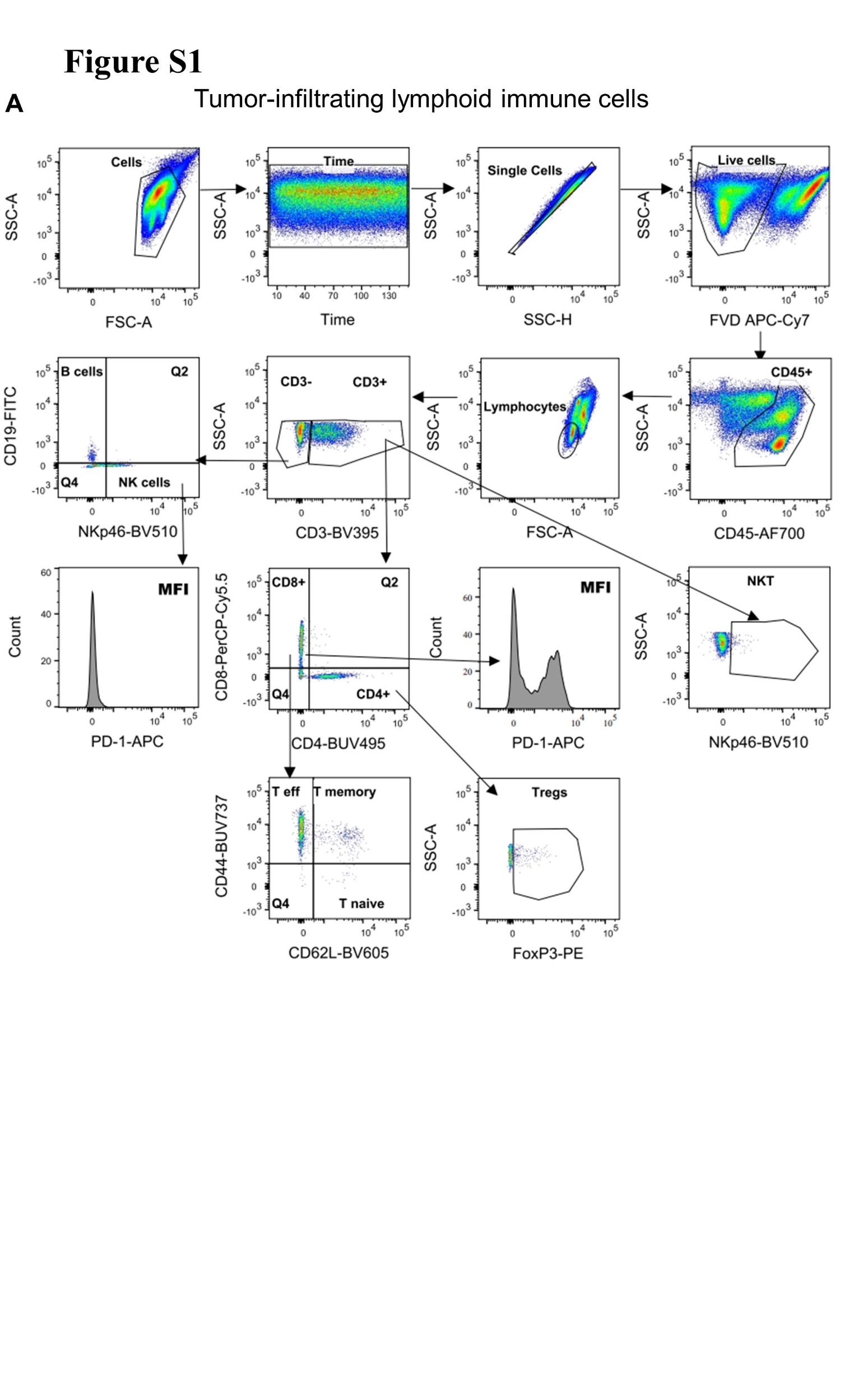


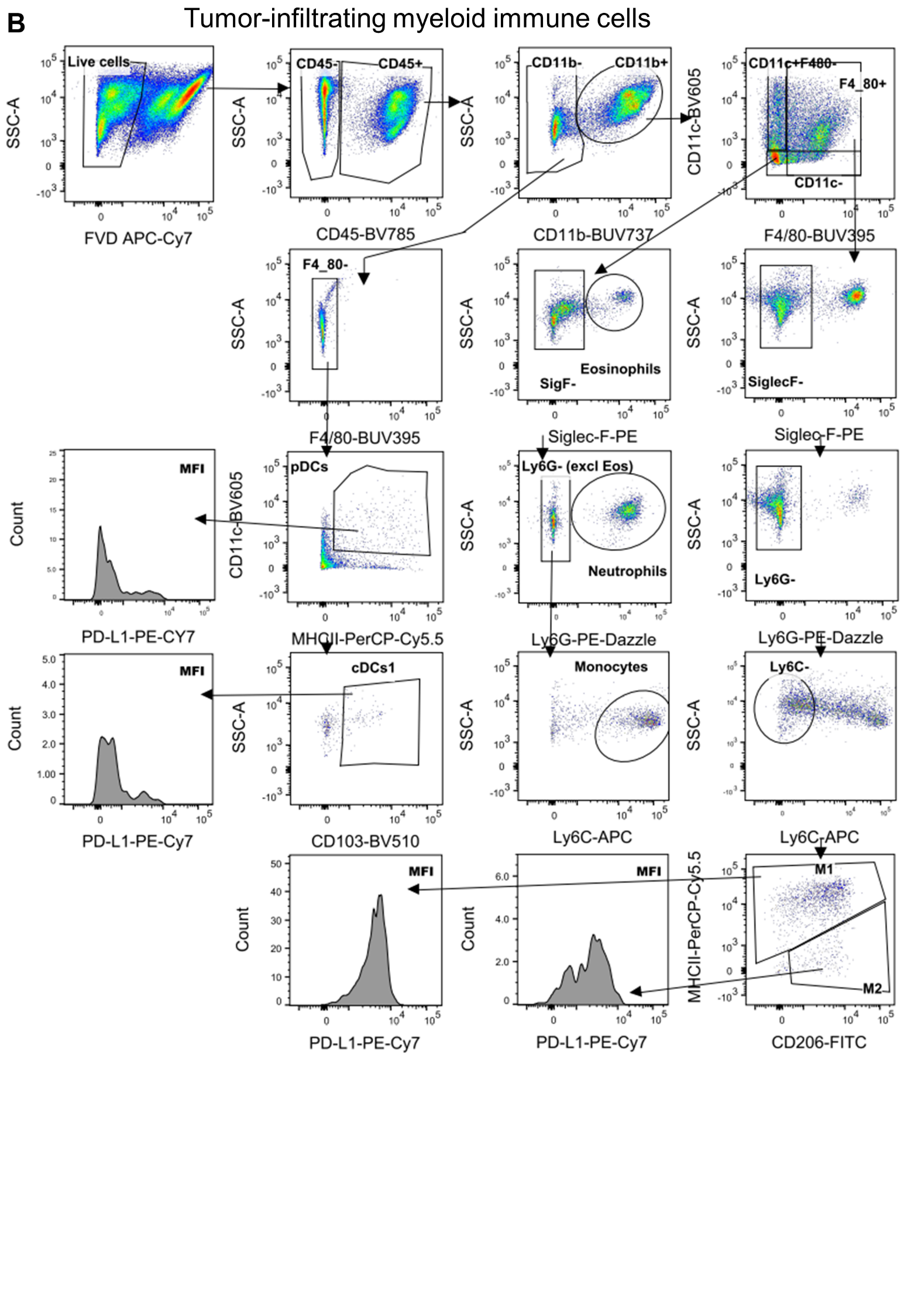


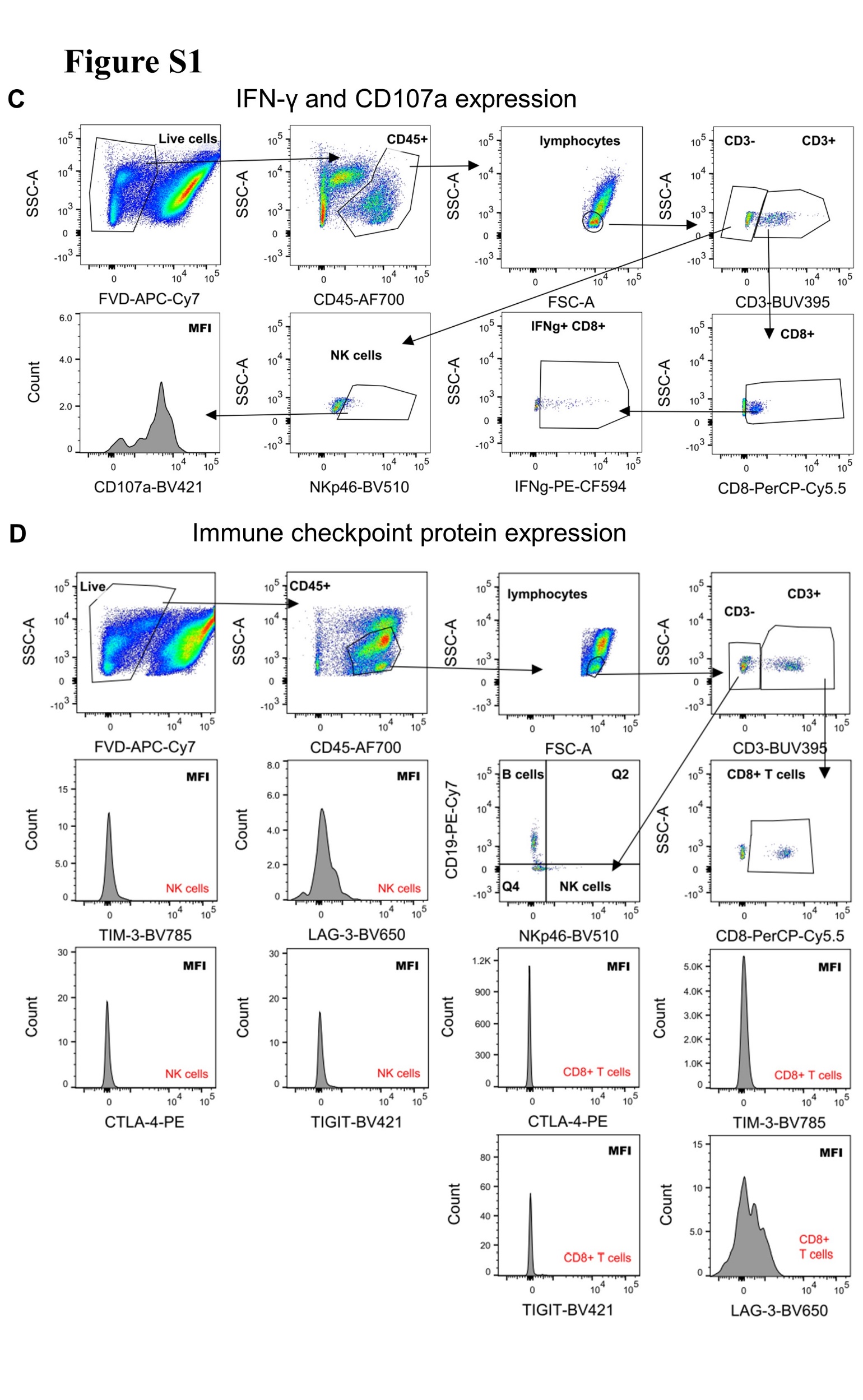


**
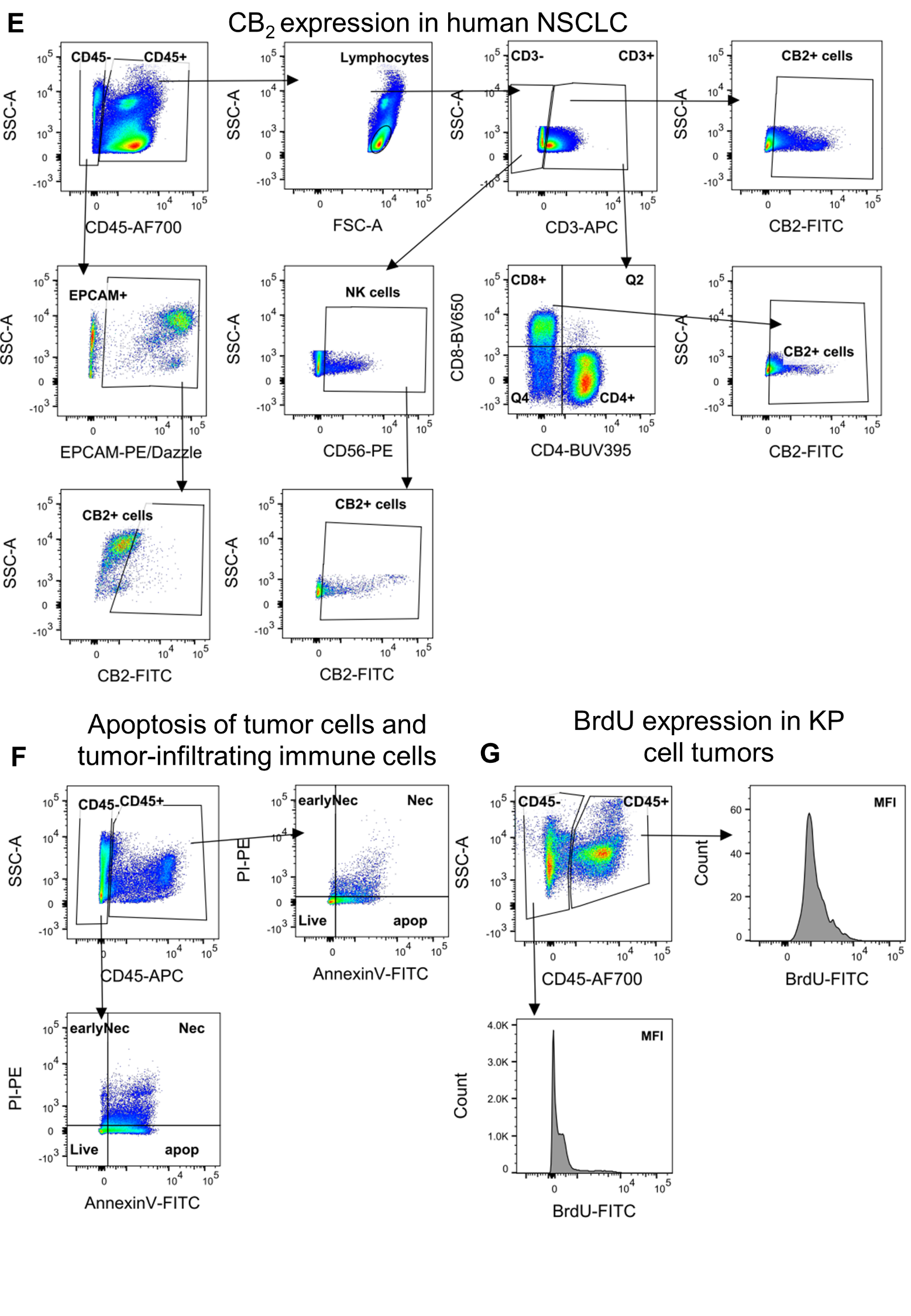
**

**FIGURE S1 |** Flow Cytometry Gating Strategies**.** Schematic representation of the flow cytometric gating strategies to analyze single cell suspensions obtained from KP cell tumors and human lung tumors. Infiltrating CD45^+^ were pre-gated for time and single cells. Dead cells were excluded and to exclude myeloid cells from the lymphoid panel an additional lymphocyte gate was used. **(A)** T cells were gated as CD45^+^/CD3^+^; NK cells as CD45^+^/CD3^-^/NKp46^+^; B cells as CD45^+^/CD3^-^/CD19^+^; CD8^+^ T cells as CD45^+^/CD3^+^/CD8^+^; CD4^+^ T cells as CD45^+^/CD3^+^/CD4^+^; T regulatory cells (Tregs) as CD45^+^/CD3^+^/CD4^+^/FoxP3^+^; CD8^+^ T effector (T eff) cells as CD45^+^/CD3^+^/CD8^+^/CD44^+^; CD8^+^ T naïve cells as CD45^+^/CD3^+^/CD8^+^/CD62L^+^; CD8^+^ T memory cells as CD45^+^/CD3^+^/CD8^+^/CD44^+^/CD62L^+^; and NKT cells as CD45^+^/CD3^+^/NKp46^+^. PD-1 expression was identified measuring median fluorescence intensity (MFI) of CD8^+^ T and NK cells in the APC channel. **(B)** Eosinophils were gated as CD45^+^/CD11b^+^/CD11c^-^/Siglec-F^+^; neutrophils as CD45^+^/CD11b^+^/CD11c^-^/Siglec-F^-^/Ly6G^+^; monocytes as CD45^+^/CD11b^+^/CD11c^-^/Siglec-F^-^/Ly6G^-^/Ly6C^+^ and macrophages as CD45^+^/CD11b^+^/CD11c^-/+^/Siglec-F^-^/Ly6G^-^/Ly6C^-^/F4/80^+^. PD-L1 expression was determined measuring MFI of M1, M2 macrophages, pDCs and cDC1s in the PE-Cy7 channel. **(C)** IFN-γ^+^ CD8^+^ T cells were gated as CD45^+^/CD3^+^/CD8^+^/IFN-γ^+^. CD107a expression was obtained measuring MFI of NK cells in the BV421 channel. **(D)** CTLA-4 expression was determined measuring MFI of CD8^+^ T and NK cells in the PE channel; TIGIT MFI of CD8^+^ T and NK cells in the BV421 channel; TIM-3 MFI of CD8^+^ T and NK cells in the BV785 channel; LAG-3 MFI of CD8^+^ T and NK cells in the BV650 channel. **(E)** CB_2_^+^ EPCAM^+^ cells were gated as CD45^-^/ EPCAM^+^/CB_2_^+^; CB_2_^+^ CD3^+^ T cells as CD45^+^/CD3^+^/CB_2_^+^; CB_2_^+^ CD8^+^ T cells as CD45^+^/CD3^+^/CD8^+^/CB_2_^+^; CB_2_^+^ NK cells CD45^+^/CD3^-^/CD56^+^/CB_2_^+^. **(F)** Apoptotic CD45^+^ cells were gated as CD45^+^/Annexin V^+^; apoptotic CD45^-^ cells as CD45^-^/Annexin V^+^. **(G)** BrdU^+^ CD45^+^ cells were gated as CD45^+^/ BrdU^+^; BrdU^-^ CD45^-^ cells as CD45^-^/ BrdU^-^. BrdU expression was identified measuring MFI of CD45^+^ and CD45^-^ cells in the FITC channel.


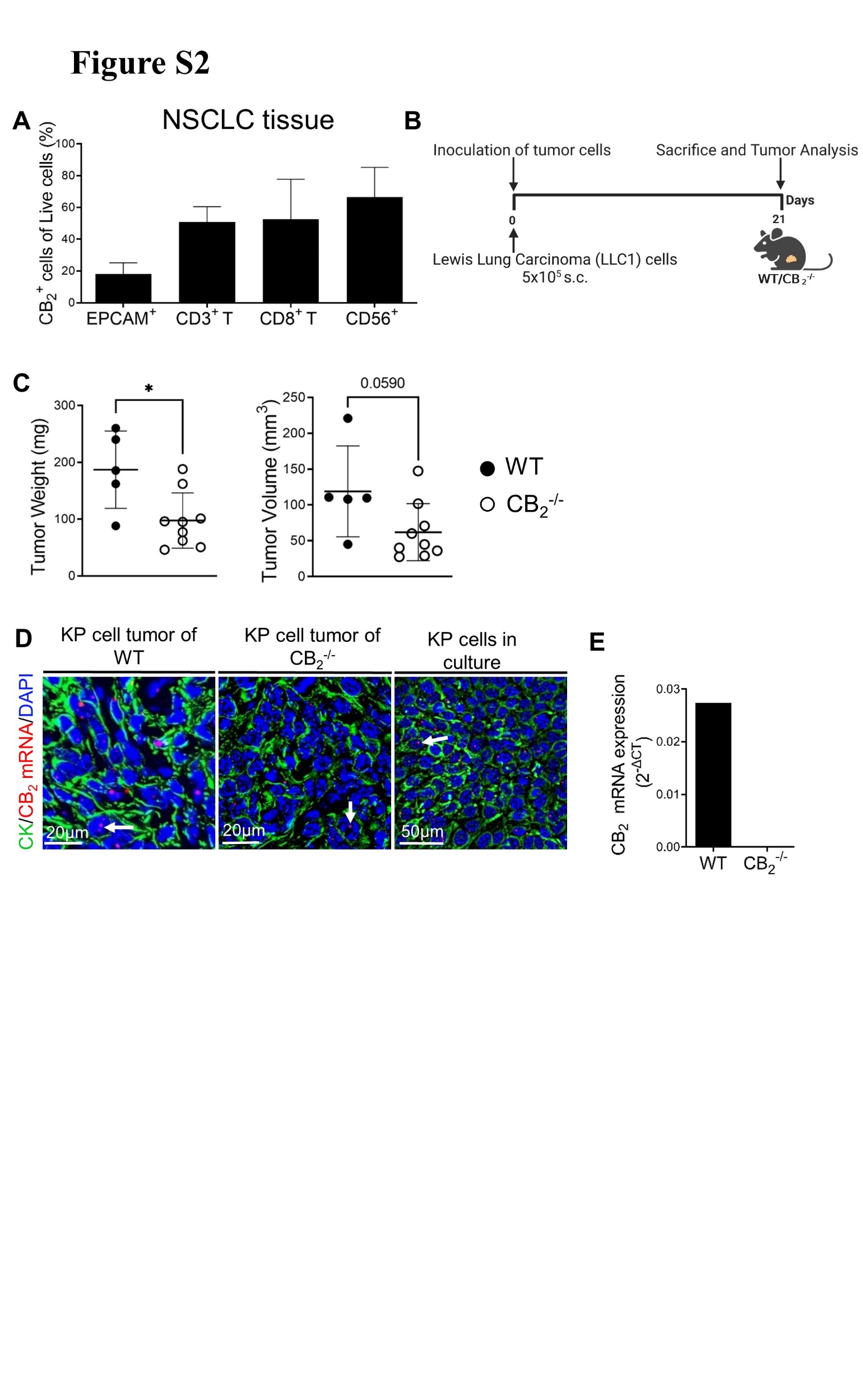
**FIGURE S2** | **(A)** Flow cytometric analysis of single cell suspensions from human NSCLC tissues reveals CB_2_ expression on cancer cells (EPCAM^+^) and tumor-infiltrating immune cells (CD3^+^ T, CD8^+^ T, and CD56^+^ NK cells). Results are shown as mean values +SD. n=4 (tissues from four different patients with NSCLC were used for analysis). **(B)** Experimental design: CB_2_^-/-^ mice and wild type (WT) littermates were subcutaneously (s.c.) injected with 5x10^5^ Lewis Lung Carcinoma (LLC1) cells on day 0. **(C)** Experimental endpoint (day 21), tumor weight and volume were measured *ex vivo*. Data indicate mean values ± SD; n=5-9. Statistical differences were evaluated by using unpaired student`s *t*-test; *p<.05. **(D)** ISH-IF staining of KP cell tumors from WT and CB_2_^-/-^ mice as well as cultured KP cells. Calibration bars are depicted on the images. Arrows denote co-localization of CB_2_ mRNA with cytokeratin-stained (CK^+^) cells. **(E)** CB_2_ primer specificity is shown via relative CB_2_ mRNA expression in lysates of healthy spleen of CB_2_^-/-^ mice and WT littermates. *NSCLC*, non-small cell lung cancer; *EPCAM*, epithelial cell adhesion molecule; *NK*, natural killer cells; *ISH-IF*, in situ hybridization and immunofluorescence; *WT*, wild type.


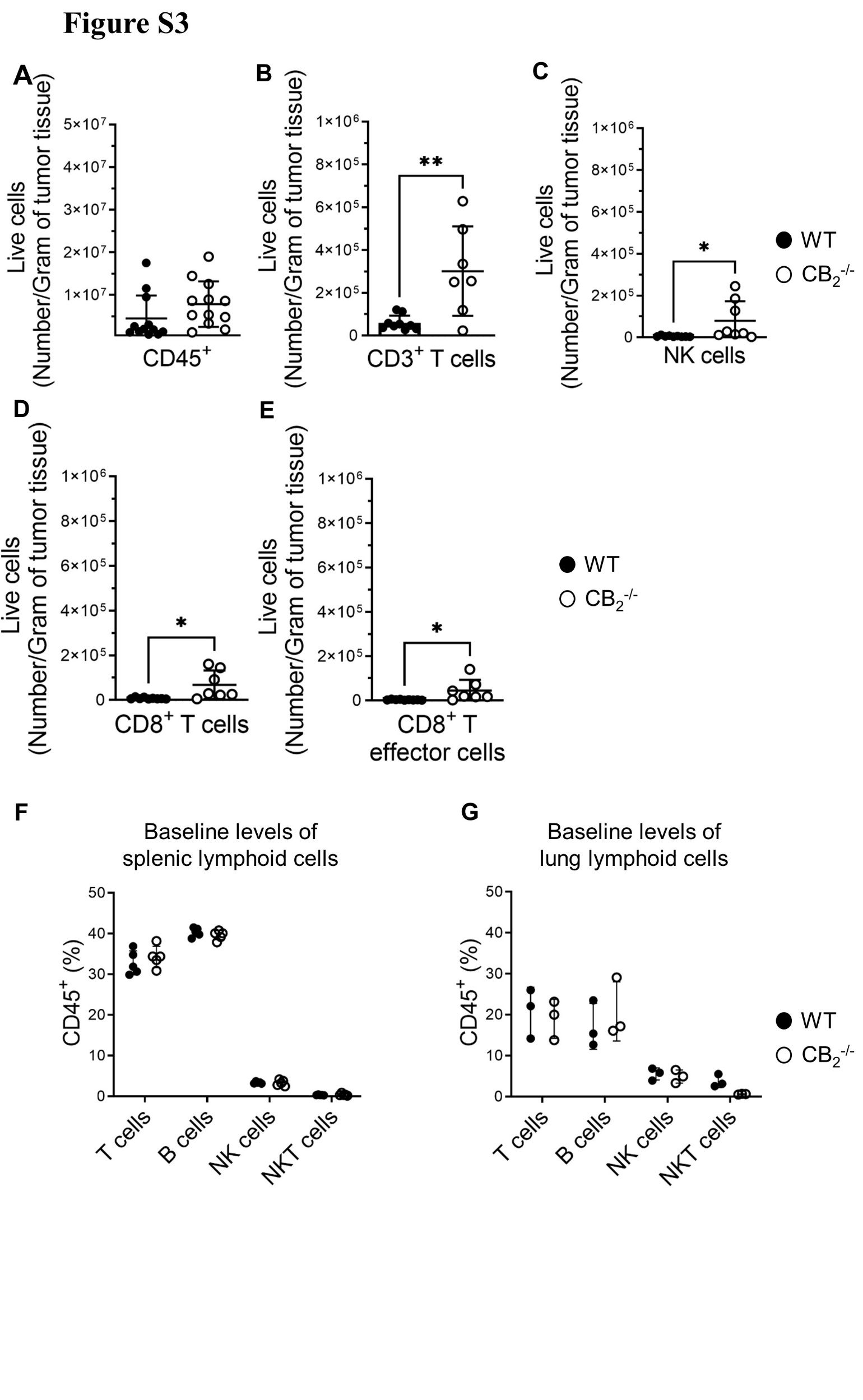


**FIGURE S3** | Deletion of CB_2_ in tumor-infiltrating immune cells provides an anti-tumorigenic immune cell profile. **(A-E)** Absolute numbers of CD45^+^ cells, CD3^+^ T cells, NKp46^+^ NK cells, CD8^+^ T cells, and CD8^+^ T effector cells (out of live cells) per gram of KP cell tumor tissue are shown. Detailed information on immune cell markers is provided in Supplemental Figure 1. Data indicate mean values ± SD. n≥7. Statistical differences were evaluated by using unpaired student`s *t*-test **(A-E)**. *p<.05; **p<.01. **(F-G)** Baseline levels of lymphoid immune cells in the spleens and lungs of CB_2_^-/-^ and WT mice. Flow cytometric analysis of single cell suspensions from spleens **(F)** and lungs **(G)** of healthy CB_2_^-/-^ and WT mice is shown. The percentages of T (CD45^+^/CD3^+^) cells, B (CD45^+^/CD3^-^/CD19^+^) cells, NK (CD45^+^/CD3^-^/CD19^-^/NKp46^+^), and NKT (CD45^+^/CD3^+^/ NKp46^+^) cells (out of CD45^+^ cells) are depicted **(F-G)**. Data indicate mean values ± SD; n=5/group **(F)**, n=3/group **(G)**. Statistical differences were evaluated by using multiple *t*-tests **(F-G)**. *NK*, natural killer cells; NKT, natural killer T cells; *WT*, wild type.

**
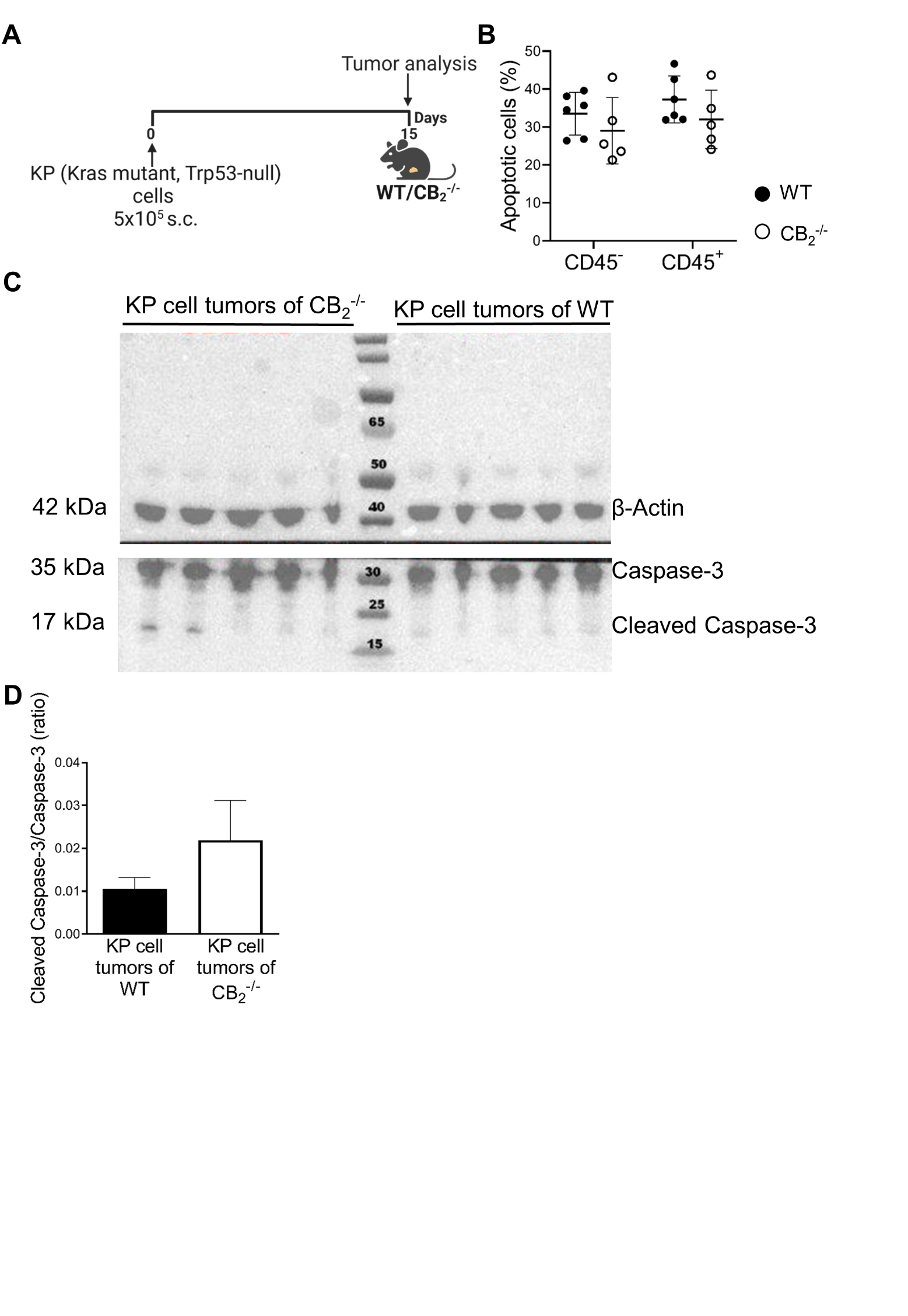
**

**FIGURE S4** | Apoptosis of tumor cells and tumor-infiltrating immune cells. **(A)** Experimental design: CB_2_^-/-^ mice and wild type (WT) littermates were subcutaneously (s.c.) injected with 5x10^5^ KP (Kras mutant, Trp53-null) lung adenocarcinoma cells on day 0. Tumor weight and volume were measured at the end of the experiment *ex vivo* on day 15. Then, tumors were collected to assess apoptosis using **(B)** flow cytometry and **(C-D)** Western blotting (WB). **(B)** Flow cytometric analysis of the cells of the tumor microenvironment (TME) revealed no significant differences in the percentage of apoptotic tumor cells (CD45^-^ cells) and tumor-infiltrating immune cells (CD45^+^ cells) between tumors from CB_2_^-/-^ and WT mice. Data indicate mean values ± SD. One representative experiment is shown. n= 5-6. **(C-D)** WB analysis of protein extracts from KP cell tumors of CB_2_^-/-^ mice and WT mice using caspase-3/cleaved caspase-3 and β-actin antibodies. Data indicate mean values ± SEM. One representative experiment is shown. n= 5/group. Statistical differences were evaluated by using unpaired student`s *t*-test **(D)**, multiple *t*-tests **(B)**.

**
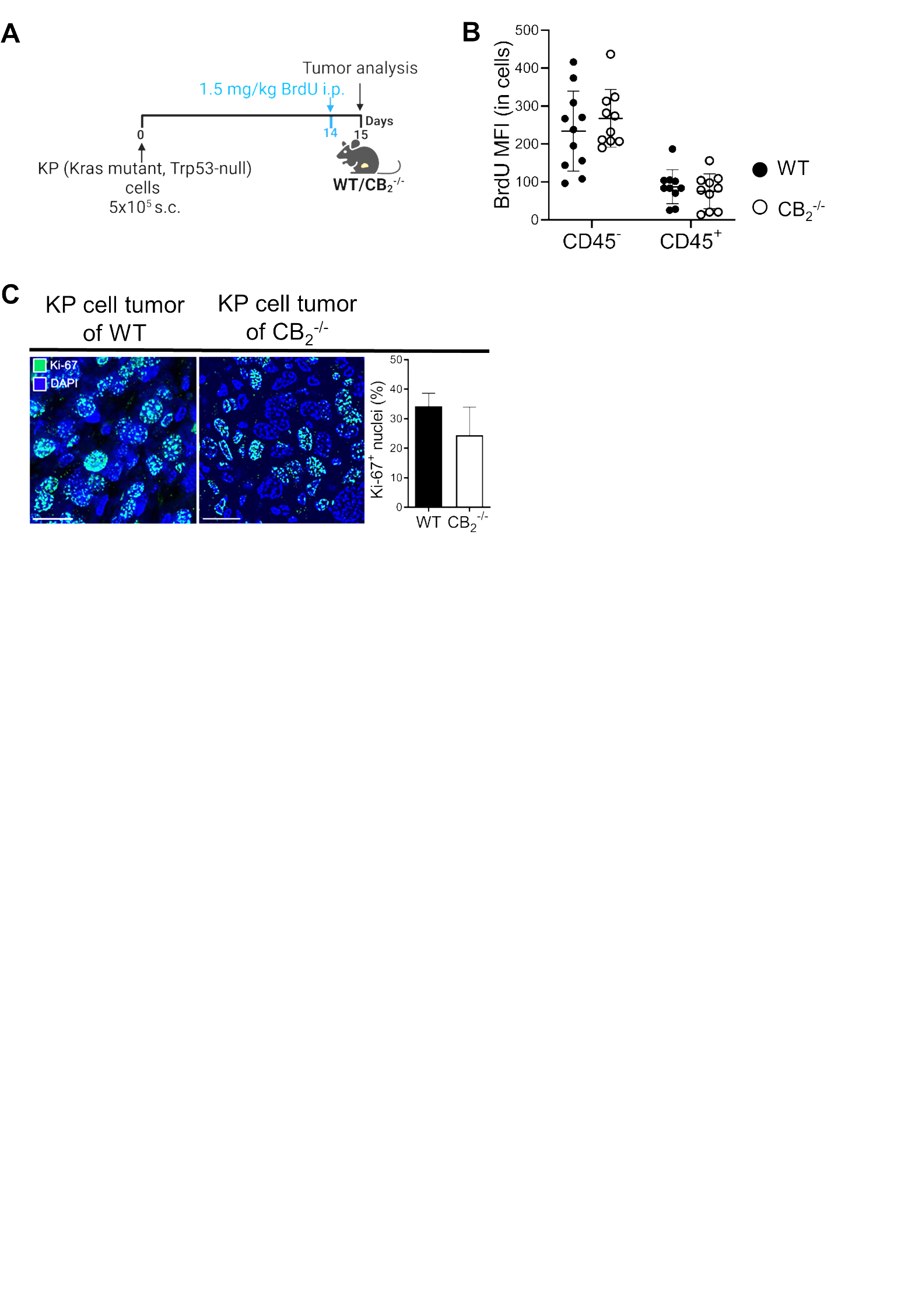
**

**FIGURE S5** | Proliferation of tumor cells and tumor-infiltrating immune cells. **A)** Experimental design: CB_2_^-/-^ mice and wild type (WT) littermates were subcutaneously (s.c.) injected with 5x10^5^ KP (Kras mutant, Trp53-null) lung adenocarcinoma cells on day 0. On day 14, mice were injected intraperitoneally (i.p.) with 1.5 mg/kg of bromodeoxyuridine (BrdU) solution to assess proliferation of tumor cells (CD45^-^ cells) as well as the cells of the TME (CD45^+^ cells) *in vivo*. On day 15, tumors were collected to perform proliferation analysis using flow cytometry. **(B)** Median fluorescence intensity (MFI) of BrdU in CD45 negative (CD45^-^) and positive cells (CD45^+^) is depicted. Data indicate mean values ±SD. One representative experiment is shown. n≥10. **(C)** Immunofluorescence staining was performed on tissue sections of KP cell tumors of WT and CB_2_^-/-^ mice to visually demonstrate proliferation of the cells using Ki-67 proliferation marker. Calibration bars=20 μm. Data indicate mean values + SD. n=3 (sections from three different tumors, 30-125 cells were counted per section). Statistical differences were evaluated by using multiple *t*-tests **(B)** and unpaired student`s *t*-test **(C)**.

**
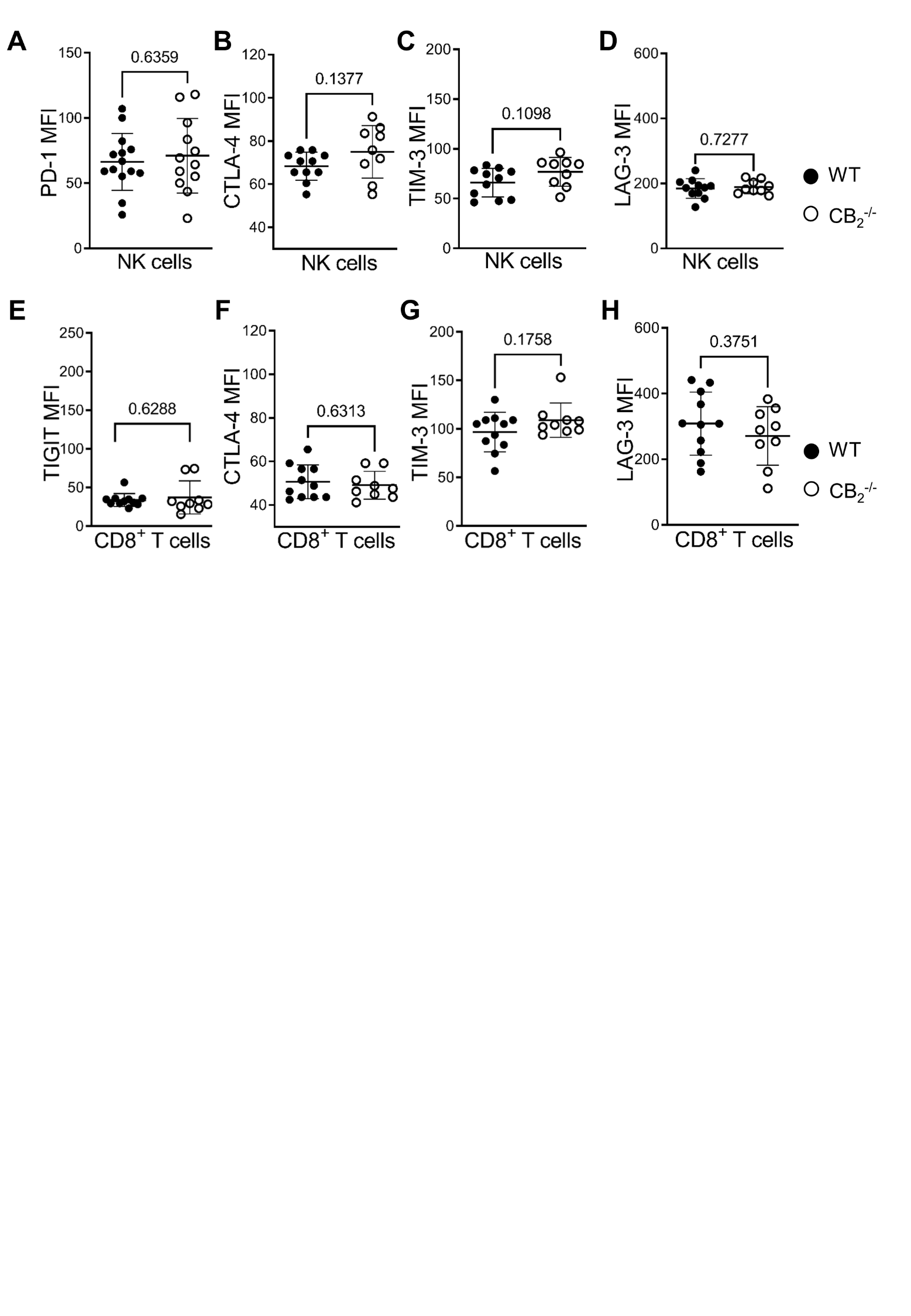
**

**FIGURE S6** | Expression of immune checkpoint proteins on tumor-infiltrating NK and CD8^+^ T cells. Flow cytometric analysis showing MFI of immune checkpoint proteins on tumor-infiltrating NKp46^+^ NK **(A-D)** and CD8^+^ T **(E-H)** cells. Detailed information on immune cell markers is provided in Supplemental Figure 1D. Data indicate mean values ±SD from two pooled independent experiments. n=9-14. All statistical differences were evaluated by using unpaired student`s *t*-test. *MFI*, median fluorescence intensity; *NK*, natural killer cells; *PD-1*, programmed death-1; *CTLA-4*, cytotoxic T-lymphocyte antigen-4; *TIM-3*, T cell immunoglobulin and mucin domain-containing protein-3; *LAG-3*, lymphocyte activation gene-3; *TIGIT*, T cell immunoglobulin and ITIM domain; *WT*, wild type.

Whole WB membranes used in Supplementary figure S4C and areas that have been cropped


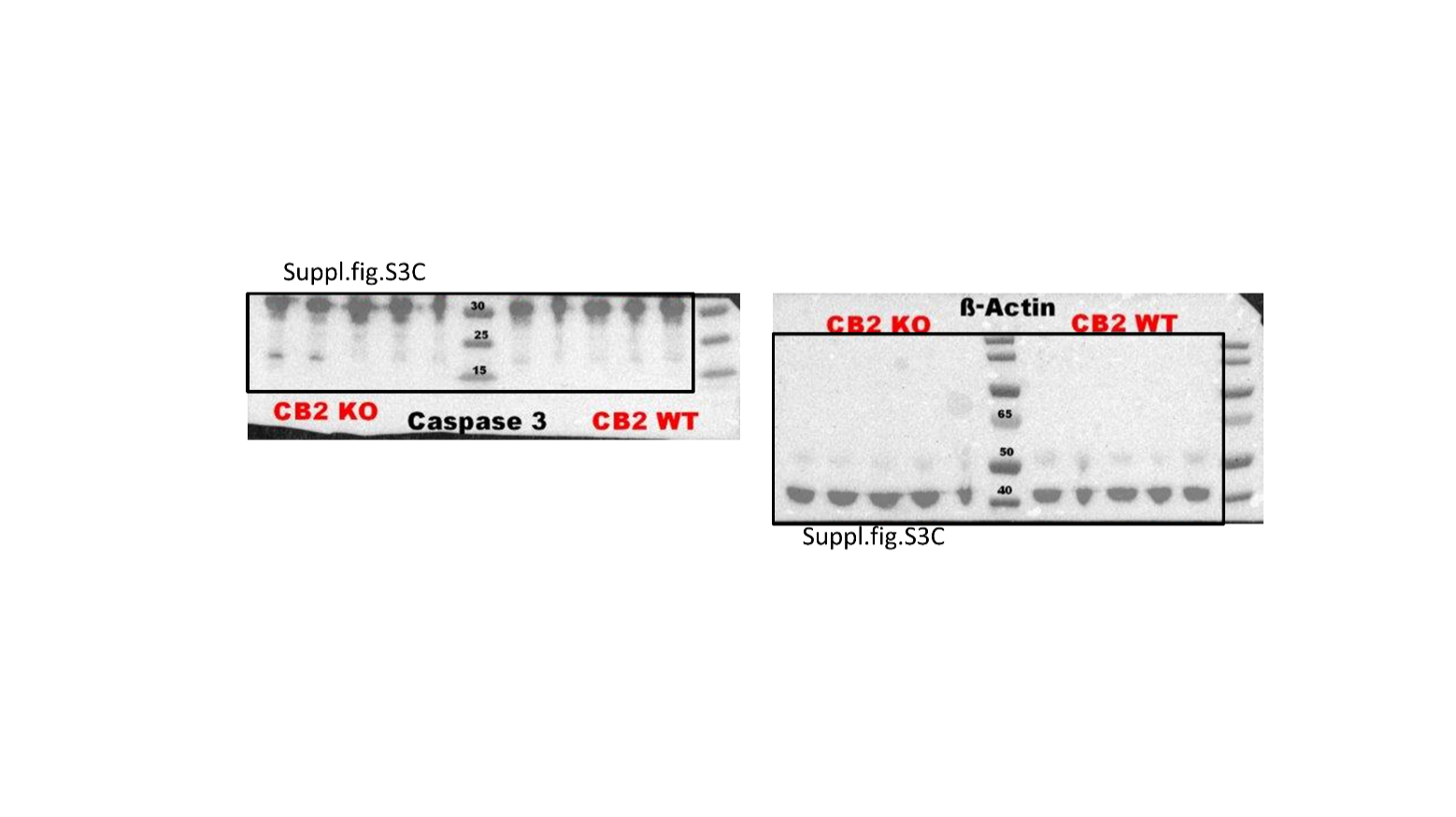

Supplement: Supplementary file 1 [file DataSheet_1.docx]
